# The Role of Tau in Neurodegenerative Diseases and Its Potential as a Therapeutic Target

**DOI:** 10.6064/2012/796024

**Published:** 2012-12-19

**Authors:** Michael S. Wolfe

**Affiliations:** Brigham and Women's Hospital, Harvard Medical School, 77 Avenue Louis Pasteur, H.I.M. 754, Boston, MA 02115, USA

## Abstract

The abnormal deposition of proteins in and around neurons is a common pathological feature of many neurodegenerative diseases. Among these pathological proteins, the microtubule-associated protein tau forms intraneuronal filaments in a spectrum of neurological disorders. The discovery that dominant mutations in the *MAPT* gene encoding tau are associated with familial frontotemporal dementia strongly supports abnormal tau protein as directly involved in disease pathogenesis. This and other evidence suggest that tau is a worthwhile target for the prevention or treatment of tau-associated neurodegenerative diseases, collectively called tauopathies. However, it is critical to understand the normal biological roles of tau, the specific molecular events that induce tau to become neurotoxic, the biochemical nature of pathogenic tau, the means by which pathogenic tau exerts neurotoxicity, and how tau pathology propagates. Based on known differences between normal and abnormal tau, a number of approaches have been taken toward the discovery of potential therapeutics. Key questions still remain open, such as the nature of the connection between the amyloid-**β** protein of Alzheimer's disease and tau pathology. Answers to these questions should help better understand the nature of tauopathies and may also reveal new therapeutic targets and strategies.

## 1. Introduction

### 1.1. Protein Aggregation in Neurodegenerative Diseases

Cells and organisms devote considerable time and energy toward the proper folding of proteins upon biosynthesis from ribosomes and prevention of inappropriate protein aggregation [1, 2]. Nevertheless, misfoldings and aggregations do occur, with some proteins being more susceptible than others. Such events would quickly cause problems without means of identifying misfolded and aggregated proteins and clearing them out. Despite the multiple means of chaperoning and correctly folding proteins and clearing out misfolded and aggregated ones, a variety of protein folding disorders can develop in humans as well as other organisms. One example is sickle cell anemia, in which a single missense mutation in the *β*-globin chain of haemoglobin leads to noncovalent polymerization and dysfunctional red blood cells [3]. Another is transthyretin amyloidosis, in which mutant transthyretin, a protein that normally transports thyroid hormone and vitamin A, aggregates in the form of amyloid deposits in the heart, kidney, nerves and intestines [4].

Neurons can be particularly sensitive to the effects of misfolded, aggregated proteins, because these cells are often in place for life after they become fully differentiated and postmitotic. Furthermore, their narrow axonal projections can be quite long, providing many opportunities to clog up the system and interfere with essential transport of biomolecules and organelles. Once axons become blocked by aggregated proteins, neurons can then degenerate from axon to cell body. Thus, the unique biology of neurons is likely the reason why abnormal protein deposition is a common feature in a variety of neurodegenerative diseases [5]. These include Alzheimer's disease (AD), characterized by the extraneuronal deposition of the amyloid *β*-protein (A*β*) in the form of plaques and the intraneuronal deposition of the microtubule-associated protein tau in the form of filaments; Parkinson's disease (PD), with neuronal deposits of *α*-synuclein; Huntington's disease (HD), with cytoplasmic and nuclear deposition of the huntingtin protein and fragments thereof; amyolateral sclerosis (ALS), characterized by TAR DNA-binding protein 43 (TDP-43) deposition in ubiquitin-positive inclusions; the prion diseases, such as Creutzfeldt-Jakob disease (CJD) in humans, mad cow disease in bovines, and scrapie in sheep, which are characterized by the aggregation of extraneuronal plaques of the prion protein.

There has been considerable debate about the pathogenic nature of the protein deposits *per se*. Indeed, dissociation between protein deposition and neurodegeneration appears to be the case with HD [6] and prion diseases [7] and may well be true of other neurodegenerative diseases [5]. Nevertheless, it is now clear that abnormal forms of these proteins, if not plaques and other microscopically observable deposits, are directly involved in initiating neurodegeneration. This is because missense mutations in the genes encoding each of these proteins (or their precursors) are associated with dominant, inherited forms of their respective diseases. In all of these genetic forms of the disease, mutation of the protein leads to the characteristic pathology, neurodegeneration, and, ultimately, disease onset and progression. Why disease onset takes decades is unclear but may be due to a reduced ability to clear out misfolded and aggregated proteins with age. This might explain why sporadic Alzheimer's disease often does not present until after age 70, and its prevalence is as high as 50% among those over age 85.

### 1.2. Tau as a Common Pathological Marker in Many Neurodegenerative Diseases

Tau deposition in the form of intraneuronal filaments is found not only in AD, but in a variety of other neurodegenerative diseases collectively referred to as tauopathies [8, 9]. These include a spectrum of diseases now placed under the umbrella name of frontotemporal dementias (FTD) (e.g., Pick's disease, corticobasal degeneration, progressive supranuclear palsy, frontotemporal lobar dementia with parkinsonism linked to chromosome 17 (FTDP-17), and dementia pugilistica). FTD is the second most common form of presenile dementia after AD. Tau pathology is also often seen in some forms of PD, ALS, and prion diseases, although it is not characteristically the most common feature of these other neurodegenerative disorders.

At the microscopic level, tau deposition is readily observed *post mortem* through silver staining of brain slices [10]. Filaments are found in neuronal extensions, leading to abnormal processes referred to as neurofibrillary tangles (NFTs) by Alois Alzheimer, who first described this type of pathology in an AD brain in 1906. Although tau is normally found primarily in axons, with smaller levels in dendrites, it can form filaments in dendrites and in the neuronal cell body, suggesting missorting. Although both amyloid plaques (of A*β*) and neurofibrillary tangles (of tau) are both common pathological features in AD, the latter correlate more closely with neurodegeneration. This observation, as well as others described later, suggests that aberrant tau might mediate A*β* pathogenicity and be more proximal to neurotoxicity in the pathogenic pathway leading to AD. In genetic forms of FTD, mutations in tau clearly implicate abnormal tau as the initiator of neurodegeneration and disease [11]. 

The particular type of neurodegeneration that manifests itself is determined by the neurons and networks of neurons in which misfolded tau accumulates and aggregates. In AD, tau pathology apparently first occurs in the entorhinal cortex, and then spreading to the hippocampus and cerebral cortex [12–16]. As these brain regions play critical roles in learning and memory, the primary problem in AD is cognitive deficits. In FTD, tau generally deposits in the frontal and temporal lobes [8], regions of the brain that are important for executive functions and behavior. Thus, the first signs of FTD are typically irrational decisions and socially inappropriate behavior. However, tau pathology can also be found in other brain areas (e.g., basal ganglia and subthalamus) depending on the specific type of FTD (e.g., progressive supranuclear palsy). 

Why particular neurons are susceptible to the buildup of misfolded tau and tau aggregation is unknown. For wild-type tau, stochastic events may trigger misfolding and aggregation in a given neuron or set of neurons. However, certain mutations of tau are associated with specific forms of FTD [8], suggesting that specific neurons and neuronal networks are especially susceptible to those particular mutations. On the other hand, the same mutation (P301L) can apparently lead to corticobasal degeneration or FTDP-17 in the same family, suggesting that other genetic, epigenetic, or environmental factors may influence which neurons are affected. Regardless of why specific neurons are affected, it is critical to understand the normal role of tau and how it becomes pathogenic in order to consider strategies for prevention and treatment.

## 2. Tau Biology

### 2.1. The Tau Gene and Haplotypes

*MAPT*, the gene encoding the tau protein, located on chromosome 17q21.3, spans ~150 kb and consists of 16 exons, 11 of which are expressed in the central nervous system [17]. Two main haplotypes exist, H1 and H2, resulting from an ancient inversion of a ~900 kb region that includes the entire *MAPT* gene. Although the amino acid sequence between H1 and H2 tau is unchanged, these haplotypes do differ in a set of defined single nucleotide polymorphisms (SNPs) and a 238 bp deletion in intron 9 in H2. Interestingly, the H1 haplotype is much more diverse than is H2, which is relatively invariant and found almost entirely in those of European ancestry. While the H1 and H2 inversion is estimated to have occurred ~3 million years ago, the H2 haplotype was apparently reintroduced in the European population some 10,000–30,000 years ago. Since *Homo sapiens* and *Homo neanderthalensis* coexisted in Europe during that time, it was suggested that Neanderthals may have reintroduced H2 into the *Homo sapien* genome [18]. Recent determination of the Neanderthal genome, however, does not support this contention [19].

Although H1 has been reported to be overrepresented in Caucasian cases of progressive supranuclear palsy [20] and sporadic PD [21], the association with *MAPT per se* is unclear. Considering the diversity of the H1 haplotype, more refined analyses support a particular subhaplotype, H1c, being highly associated with PSP and CBD, with H2 being strongly negatively associated [22]. The H1c subhaplotype has also been reported to be associated with sporadic AD [23], although this has been controversial. Since the H1 and H2 haplotypes do not differ with respect to tau protein sequence, any pathogenic effects associated with a particular haplotype or subhaplotype may instead be due to differences in transcription, splicing or other posttranscriptional modifications, transcript stability or localization, or rates of translation. It is already clear that silent or intronic mutations that shift tau pre-mRNA alternative splicing can cause familial FTD (see below), and recent evidence suggests that the H1c subhaplotype is associated with a similar alteration in splicing [24, 25]. 

### 2.2. Protein Expression, Sequence, and Splice Isoforms

Tau protein is expressed in a variety of mammalian tissues, being found at relatively high levels in the heart, skeletal muscle, lung, kidney, and testis and at low levels in the adrenal gland, stomach, and liver [26]. However, it is most highly enriched in neurons in the central nervous system. This makes sense, as tau is a microtubule-associated protein [27, 28], and microtubules are especially important to neuronal structure and function. The tau protein is primarily found within axons [29], although it is also found at low levels normally in dendrites [30]. Abnormal accumulation of tau in somatodendritic compartments is associated with Alzheimer's and other tauopathies [31].

**Figure 1:**
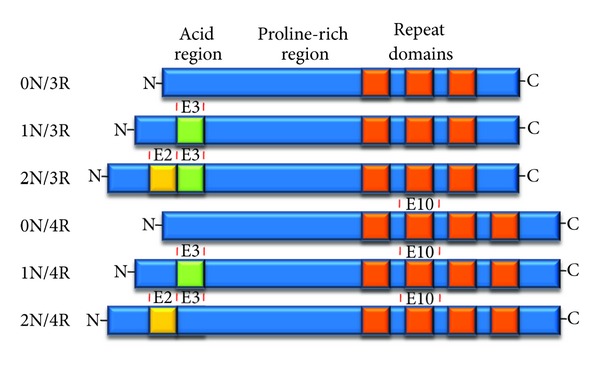
Domains and splice isoforms of tau. Exons 2, 3 and 10 are alternatively spliced. Inclusion/exclusion of exons 2 and 3 are denoted as 0N, 1N, or 2N. Inclusion/exclusion of exon 10, encoding the second of four possible repeated microtubule binding domains, are denoted as 3R or 4R.

The tau protein contains several imperfectly repeated 18-residue microtubule-binding domains in the C-terminal region [32–34] (Figure 1). These are linked through a proline-rich region to an acidic N-terminal region (also called the projection domain) [35]. The tau pre-mRNA undergoes alternative splicing at exons 2, 3, and 10 to give six different possible isoforms [36]. Exons 2 and 3, encoding 29-residue stretches near the N-terminus, undergo coupled splicing, with exon 2 inclusion being dependent on exon 3 inclusion. Variations in exon 2/3 splicing are referred to at 0N (neither included), 1N (exon 3 included), or 2N (both exons 2 and 3 included). Exon 10 encodes 31 amino acids and the second of four possible microtubule binding domains. Inclusion of exon 10 generates 4-repeat or 4R tau, while exclusion forms 3-repeat or 3R tau [36–38]. A small degree of alternative splicing can also occur in exon 6, resulting in other very minor tau isoforms [39].

The expression of the different tau isoforms in the brain is under developmental control, suggesting that the regulation of tau isoforms is important during formation of the brain. Human fetal tau is almost exclusively 0N/3R tau, while adults express all six isoforms [38, 40]. Disruption of the normal 4R-to-3R ratio is associated with neurodegeneration (see the following). Consistent with an important role of tau in brain development, microdeletions in *MAPT* are associated with mental retardation [41, 42], and knockdown of tau can result in reduced neuronal connectivity in the brain [43]. Despite these observations, complete knockout of tau in mice yields viable and fertile animals with grossly normal brain anatomy and no evidence of neurodegeneration [44–48], although this may be due to functional redundancy with other microtubule-associated proteins (MAPs). Of four independent tau knockout lines that have been established so far, only one has been reported to have motor deficits and hyperactivity, with debatable learning impairments [46]. No deficits of any kind have been reported in hemizygous knockout mice. 

### 2.3. Phosphorylation

**Figure 2:**
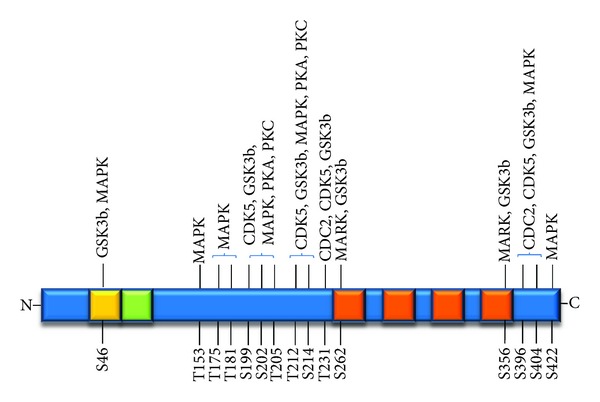
Sites of phosphorylation in tau and the kinases implicated.

The tau protein can undergo a variety of phosphorylation events at its many serine, threonine, and tyrosine residues, although most occur at Ser/Thr-Pro sites [49]. Some 30 or more possible phosphorylation sites are found in every tau isoform. These phosphorylations can control the normal biological functions of tau, such as its role in microtubule stability, as well as its pathological functions, such as its ability to self-assemble into neuronal filaments found in neurodegenerative diseases. The functions, if any, either physiological and pathological, of many of these phosphorylations are unknown, and in some cases it is unclear if they even occur *in vivo*. Moreover, these phosphorylations can be mediated by numerous candidate kinases *in vitro*, but the ability of many of them to carry out the specific phosphorylations of tau in cells and *in vivo* at physiological levels and locations is likewise unclear. A diagram showing sites on tau that have been reported to be phosphorylated is depicted in Figure 2.

Some of these phosphorylations, however, do appear to be particularly relevant. Among these are Ser202, Thr205, Ser396, Ser404, as well as repeated KXGS motifs located within each microtubule binding domain. The microtubule-affinity-regulating kinase (MARK) selectively phosphorylates the KXGS motifs, particularly the one including Ser262 [50, 51]. Phosphorylations at these sites reduce the affinity of tau for microtubules and may thereby regulate microtubule formation and function [52, 53]. The glycogen synthase kinase 3*β* (GSK3*β*) and cyclin-dependent kinase 5 (cdk5) are also associated with many of tau phosphorylations, both *in vitro* and *in vivo*, although the ability of cdk5 to do so directly *in vivo* is unclear [54, 55]. Cdk5 is activated by the noncyclin regulatory proteins p39 and p35 in postmitotic cells; however, proteolysis of p39 and p35 by calpain to p29 and p25, respectively, results in prolonged half-life of these regulatory proteins and, therefore, prolonged cdk5 activity and increased tau phosphorylation. Protein kinase A is also especially implicated in tau phosphorylations [56, 57]. Although tau is hyperphosphorylated in AD, it is unclear which particular sites are relevant, or even whether hyperphophorylation occurs before or after filament formation. Tau can also undergo dephosphorylation by phosphatases such as PP1 and PP2A. The balance between kinase activity and phosphatase activity is critical to the phosphorylation state of tau and therefore to tau function and dysfunction.

### 2.4. Role of Tau in Microtubule Formation, Stability, and Dynamics

The most studied cellular role of tau is in its interaction with and effects on microtubules. While microtubules are most noted in cell biology for the formation of the mitotic spindle during cell division, these polymers of tubulin are essential to postmitotic cells as well. Microtubules are particularly important for neuronal integrity and function, providing structure to the axonal processes that extend from the neuronal cell body and a path for the transport of materials to and from the often relatively distant synapses. Tau is involved in the assembly and stability of microtubules [27, 28], roles that have been extensively studied; however, *in vivo*, tau may be more involved in microtubule dynamics, in the assembly/disassembly process critical to axonal transport. In addition, the gradient of tau along the axon, with levels highest at the synapse [58], likewise suggests a role in axonal transport, and tau can block the binding of motor proteins that move materials along microtubules [59, 60]. Thus, the high levels of microtubule-bound tau near synapses may favor the release of motor proteins and their cargo where needed.

As mentioned earlier, despite these seemingly critical functions of tau, tau knockout mice are viable, fertile, and relatively normal, with no signs of neurodegeneration. Furthermore, knockdown of tau with siRNA does not kill primary neurons in culture or prevent axon formation [61]. Thus, tau is not essential to neurons or to microtubules, likely due to compensation by and/or redundant functions of other proteins. Indeed, the microtubule-associated protein 1B (MAP1B), also found primarily in axons, apparently plays such roles. MAP1B is likewise involved in microtubule stability, and knockout of MAP1B results in abnormal brain development and early death [62]. Knockout of both tau and MAP1B worsens this phenotype [62]. However, to determine if MAP1B or other factors compensate for the loss of tau during development, conditional tau knockout mice should be generated in order to abrogate tau in the adult mouse brain. Addressing this issue is critical for determining how misfolded or aggregated tau might cause neurodegeneration (discussed in more detail later).

### 2.5. Other Tau Functions

Tau has been reported to interact with a number of other proteins besides tubulin/microtubules [63], although the biological relevance of many of these interactions is not clear. However, in recent years evidence has been mounting that the interaction between tau and the protein kinase Fyn may be important for modulating neuronal cell signaling through Fyn [30]. As mentioned earlier, tau is found normally at low levels in dendrites, colocalizing with Fyn and the postsynaptic density protein 95 (PSD95). These three proteins can be coimmunoprecipitated, suggesting that tau may be serving as a scaffolding protein along with PSD95 to localize Fyn at synapses, enabling Fyn activation through NMDA receptors. Indeed, tau is required for phosphorylation of NMDA receptor subunit NR2B in dendrites. The tau-Fyn interaction may also play a role in process extension in oligodendrites [64].

## 3. Tau in Neurodegenerative Diseases

### 3.1. Alzheimer's Disease

#### 3.1.1. Amyloid and Tau

**Figure 3:**
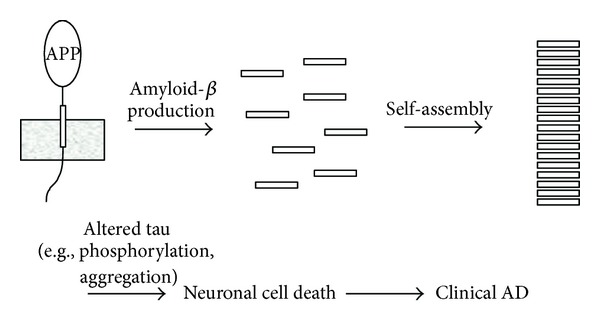
The amyloid hypothesis of Alzheimer's disease and the role of tau. Evidence supports the formation and aggregation of the amyloid *β*-protein as the initiator of the disease process, with tau playing a subsequent role. Possible A*β*-induced pathological changes in tau include hyperphosphorylation or aggregation.

The AD brain is characterized by extraneuronal deposits of A*β* and intraneuronal filaments of tau [10]. A*β* is produced through proteolytic processing of the type I integral membrane protein amyloid *β*-protein precursor (APP) [65] (Figure 3). The membrane-tethered *β*-secretase sheds the APP ectodomain [66], followed by cleavage of the 12-kDa remnant within its transmembrane domain by the *γ*-secretase complex [67]. Dominant missense mutations that cause early-onset, familial AD are found in the *APP* gene, in and around the small (4 kDa) A*β* region of the much larger APP, and these mutations affect the level, type, or aggregation properties of A*β* produced [68]. Dominant missense mutations are also found in *Presenilin*, the protein of which is the catalytic component of *γ*-secretase, and these mutations also change the type of A*β*, increasing the proportion of longer, more aggregation-prone forms of the protein [68]. No mutations in *MAPT* have been found associated with AD. Rather, such mutations are associated with FTD (discussed later). Thus, human genetics supports A*β* as the initiator of AD pathogenesis [68]. However, tau is a common feature of a spectrum of neurodegenerative diseases, and A*β* may be one of many possible ways of triggering tau pathology [9].

Further evidence that tau is downstream of A*β* but critical to AD pathogenesis comes from mouse studies. Mice that are doubly transgenic for human APP and presenilin-1 carrying familial AD mutations display amyloid plaques pathology and learning and memory deficits [69]. Crossing these mice with tau knockout mice revealed that loss of tau did not affect APP expression, A*β* production, or plaque formation [47]. However, the cognitive deficits were rescued in a gene-dosage-dependent manner, with loss of one tau allele providing partial rescue. In addition, a seizure phenotype observed in the transgenic mice was likewise rescued. Surprisingly though, in another report, crossing a tau knockout with a human mutant APP mouse led to increased axonal degeneration [70]. Mice transgenic for human tau can show tau pathology and cognitive and behavioral deficits [9]. However, crossing these mice with the APP/Presenilin-1 mice resulted in triple transgenic mice with increased tau pathology, along with amyloid pathology, and more severe learning and memory deficits [71]. Treatment of the triple transgenic mice with anti-A*β* monoclonal antibodies attenuated the tau pathology as well as the cognitive deficits [72]. Overall, these findings suggest that tau is part of the pathogenic process subsequent to A*β* and might be a worthwhile target for therapeutic intervention.

Cellular studies also support the ability of A*β* to affect tau. Treatment of neuronal cultures with A*β* can lead to hyperphosphorylation of tau [73, 74], although this has not been observed in APP transgenic mice with endogenous tau expression. Perhaps due to the increase in tau phosphorylation and reduced affinity of tau for microtubules, A*β* treatment can also induce the inappropriate increase of tau in dendritic spines, a phenomenon that correlates with a degree of spine degeneration [74]. Most recently, dimeric A*β* isolated from *postmortem* human AD brains has been reported to cause tau hyperphosphorylation in cultured rat hippocampal neurons, release of tau from microtubules, and neurite degeneration [75]. Moreover, A*β* oligomers containing a truncated, N-terminally pyroglutaminylated form of A*β* implicated in AD pathogenesis were found to be highly toxic to cultured neurons in a tau-dependent manner [76]. However, despite the possibility that A*β* may affect tau phosphorylation, mislocalization, and neuronal health, the ability of A*β* to cause assembly of endogenous tau into oligomers or filaments has not been observed. 

How A*β* might cause these changes in tau is not clear. However, one possible connection is through the protein tyrosine kinase Fyn, which, as mentioned earlier, can interact with tau and PSD95 in dendrites and thereby trigger phosphorylation of the NMDA receptor subunit NR2B. The cellular prion protein (PrPc) has been recently reported as a receptor for soluble A*β* oligomers [77], forms of A*β* more implicated in AD pathogenesis than amyloid plaques. This interaction can lead to increased Fyn activity and NR2B phosphorylation in neurites. Whether knockout or knockdown of tau can alter this affect of A*β* remains to be tested, but as pointed out previously, tau is required for NR2B phosphorylation by Fyn in dendrites. Further evidence for an A*β*-Fyn-tau pathogenic pathway in AD: Fyn overexpression worsens the neuronal and cognitive phenotype of APP transgenic mice [78, 79], and this effect is attenuated by knockout of tau [80].

#### 3.1.2. Loss versus Gain of Function

The question of whether aberrant tau is pathogenic due to a loss of function or a gain of function has been debated considerably over the years. On the one hand, hyperphosphorylation of tau and tau aggregation may lead to less tau that is capable of facilitating microtubule formation, stability, and dynamics. As microtubules are especially important for neuronal health, particularly in axons, reduction of functional microtubules would lead to disruption in the transport of molecules and organelles to and from the synapse, thereby resulting in synaptic dysfunction and degeneration. On the other hand, hyperphosphorylated and/or aggregated forms of tau may result in a toxic gain of function, in which the aberrant species *per se* cause neuronal dysfunction and degeneration. The aggregated forms, for instance, may block the axon to prevent proper transfer of materials along microtubules. It seems clear that pathogenesis is not strictly due to a loss of function; as the tau knockout mice do not display neurodegeneration, tau can be knocked down in neuronal cultures without causing cell death or preventing axon formation, and none of the FTD-causing MAPT mutations cause deletion or truncation of the protein. These facts, however, do not preclude that a reduction of microtubule-associated tau, due to tau hyperphosphorylation and/or aggregation, may be a secondary contributor to neuronal dysfunction and death along with the primary contributor, an aberrant gain of toxic function.

#### 3.1.3. The Spread of Tauopathy

Tau pathology in AD is first observed in the entorhinal cortex [12]. This region of the brain, located in the caudal end of the temporal lobe, is connected to the hippocampus and the neocortex and critical for memory formation and consolidation. From the entorhinal cortex, tau pathology spreads, apparently from neuron to neuron via synaptic connections, to the hippocampus, and then to the neocortex [12]. Studies in cell culture have shown that extracellular aggregates of tau can be taken up by cells through endocytosis and cause aggregation of intracellular tau that then spreads throughout the culture [81–83]. Similar experiments have been performed* in vivo*, in which brain extracts containing aggregated tau were injected into the brains of mice transgenic for human tau, leading to aggregation of tau expressed from the transgene and the spreading of tau pathology into connected regions of the brain [84].

Most recently, mice were engineered to express human P301L tau specifically in the entorhinal cortex in response to tetracycline treatment [15, 16]. This mutation causes familial frontotemporal dementia with typical tau pathology. Induction of mutant tau expression in the entorhinal cortex in adult mice resulted in tau aggregation and neurodegeneration in this specific region. From there, the pathology spread to the hippocampus and neighboring regions, areas in which only endogenous mouse tau was expressed at normal physiological levels. With the caveat that very low levels of the mutant transgenic tau might have been expressed in areas other than the entorhinal cortex, the results suggest that pathological tau can induce similar aggregated states in normal tau in neighboring or connected neurons. Such a process is reminiscent of the mechanism by which prion proteins propagate: a pathological conformation that leads to aggregation can induce normal forms of the protein to take on the same pathological conformation [86]. While tau is not infectious like pathological prion proteins, it can apparently propagate a pathological conformation from neuron to neuron in a similar manner.

How aberrant tau might spread from one neuron to another though is unclear. One possibility is that misfolded and aggregated tau is released upon neuronal cell death, whereupon it is taken up by neighboring neurons via endocytosis. Another is that tau is somehow secreted from neurons, even though it is not the kind of protein that generally enters the secretory pathway. A particularly intriguing alternative is that tau is contained within exosomes [87], microvesicles that are released from the cell when multivesicular bodies fuse with the plasma membrane. Subsequent fusion with the membrane of a neighboring neuron results in uptake. The fact that tau is released, whether by dead neurons or live, by secretion or in exosomes, is clear: tau is detectable in human cerebral spinal fluid (CSF), and an increase in CSF tau correlates with AD. Indeed, CSF tau or phosphorylated tau is a leading biomarker for prodromal AD [88].

#### 3.1.4. Oligomers versus Aggregates

For both amyloid plaques and tau filaments, a major question for many decades has been whether these microscopic deposits are involved in the pathogenesis of AD, are markers where neuronal cell death and destruction have already occurred, or are otherwise tightly coupled to the actual pathogenic forms of the proteins. The formation of insoluble aggregates of A*β* or tau could even be a protective response, reducing the levels of the real neurotoxic species and allowing the clearance of these proteins through processes such as phagocytosis or autophagy. For A*β*, this may well be the case, as amyloid deposits *per se* do not correlate well with neurodegeneration [89]. Tau filament formation and neurofibrillary tangles are more closely correlated with neurodegeneration than amyloid plaques are. For A*β*, soluble oligomeric forms of the protein, rather than insoluble deposits, have been especially implicated in AD pathogenesis [90]. As for tau, soluble oligomers at the very least appear to be on the pathway to NFTs [91–93] and may well be pathogenic themselves.

Neuronal cell death and synaptic defects have been seen independent of tau-containing NFTs in mice transgenic for wild-type human tau [94, 95]. Loss of synapses in the hippocampus, impaired synaptic function, and microgliosis also occurred before formation of NFTs in an FTD-mutant human tau mouse model [96], observations that have been reported in other tau animal models as well [97, 98]. Intriguingly, a mouse line with inducible FTD-mutant tau recovered in cognitive function and stabilized in number of neurons upon lowering transgenic expression, even though NFT formation continued [99]. Moreover, accumulation of oligomeric tau correlated better than NFTs with neurodegeneration and behavioral deficits in two different tau transgenic mouse models [100, 101]. In human AD, neuronal loss can apparently precede NFT formation [13], and granular tau oligomers have been detected and isolated at very early stages of the disease [91, 102]. Taken together, these results suggest that tau oligomer accumulation, neuronal loss, and behavioral deficits occur before the formation of NFTs. Nevertheless, it seems difficult to envision that accumulation of tau filaments in neuronal soma and processes can be consistent with a healthy, functioning neuron. Indeed, extraneuronal tau filaments are found in the shape of neuronal cell bodies, and hence their name “ghost tangles” [103]. NFTs are not likely harmless, although perhaps they are less deleterious than soluble tau oligomers. However, few studies have examined tau oligomers in AD patients. The development of tau oligomer-specific antibodies [93] may offer an important new tool to elucidate the role of soluble tau aggregates in AD pathogenesis.

#### 3.1.5. Proteolysis

The proteolytic degradation of tau has been implicated in the pathogenesis of AD. A number of different proteases have been found capable of cleaving tau, at least *in vitro* [104]. Among these proteases, caspases are perhaps the most validated. Caspases are cysteine proteases that cleavage substrates with aspartate residues in position P1. These proteases are produced as inactive zymogens, and activation of certain caspases is a key step in programmed cell death or apoptosis. Several caspases are capable of proteolyzing tau at D421 (2N/4R isoform numbering), close to the C-terminus, to generate a fragment ~5 kDa smaller. This truncation of tau has been validated both *in vitro* and *in vivo* and is found in NFTs in the AD brain, although its role and that of caspase-3 in pathogenesis are unclear [105–110]. 

Interestingly, treatment of cultured cells with A*β* leads to tau cleaved at D421, and such truncated tau can facilitate aggregation [105, 111]. Thus, a pathway has been suggested in which neurotoxic forms of A*β* can induce caspase cleavage of tau to the D421-truncated form, providing seeds for the filament formation of full-length tau. However, such aggregation of endogenous tau upon expression of truncated tau in cells has not been observed, except when coexpressed with GSK3*β* [112]. The meaning of this is unclear, as pseudophosphorylation of S422 inhibits tau cleavage at D421 *in vitro* [106]. The role of caspase cleavage of tau in neurodegeneration is further complicated by a recent *in vivo* brain imaging study in tau transgenic mice [113]. In this study, neurons with both activated caspases and NFTs were generally not undergoing apoptosis, suggesting that tau truncation by caspases may correlate with tau filament formation but not neurodegeneration. Caspase cleavage preceded NFT formation, consistent with, although not proving, a cause-and-effect relationship. Interestingly, most NFT-containing neurons did not contain active caspase.

Calpains, calcium-activated cysteine proteases, have also been particularly implicated in tau cleavage. Calpain are abnormally activated in the AD brain, and levels of endogenous calpain inhibitor calpastatin are reduced [114, 115]. The formation of a 17 kDa fragment of tau is seen both *in vivo* and *in vitro* [116, 117] and is triggered in neuronal cell culture by treatment with aggregated A*β* [118]. The generation of this 17-kDa tau fragment has been attributed to calpain, as its appearance is blocked by calpain inhibitor [118]. Although the exact identity of the fragment is unknown, expression of an approximate recombinant version, tau [45-230], can lead to apoptosis in cell culture [118]. However, the presence of the 17-kDa tau fragment has not been detected in human AD brains, raising concerns about its relevance to pathogenesis. Other proteases suggested to cleave tau but with less validation include thrombin, various cathepsins, and puromycin-sensitive aminopeptidase [104]. The proteasome has also been implicated and may be particularly important for the degradation of misfolded tau [119, 120].

### 3.2. Frontotemporal Dementia (FTD)

#### 3.2.1. Tau Mutations in FTD

In 1998 mutations in the tau gene were discovered to be associated with frontotemporal dementia with parkinsonism linked to chromosome 17 (and specifically characterized by tau pathology, now called FTDP-17T) [121–123]. Initially identified mutations cluster in and around the regions encoding the microtubule binding domains, suggesting that perturbed ability to bind microtubules might be involved in neuronal destruction and death in these families. The FTDP-17T mutations provided clear evidence that alterations in tau alone are sufficient to cause neurodegenerative disease and strongly suggested that aberrant tau plays a pathogenic role in other tauopathies, including AD. 

**Figure 4:**
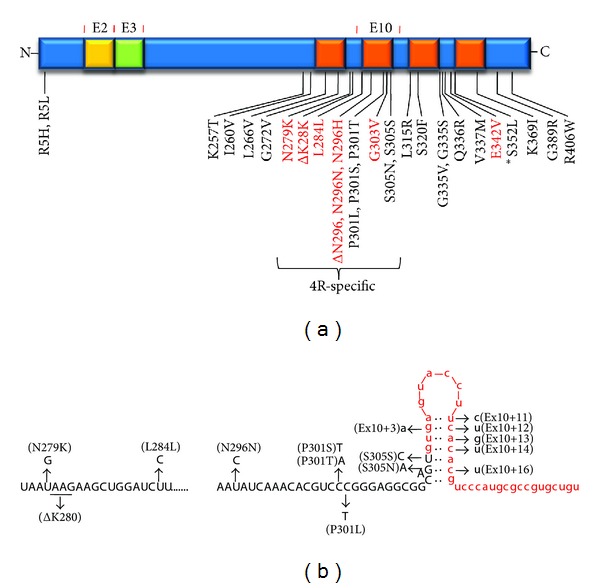
Mutations in tau associated with dominant familial frontotemporal dementia. (a) Almost all mutations in the coding region are found in the C-terminal region, in and around the microtubule binding domains. Some of these are specific to the 4R isoforms, as they are encoded in exon 10. Mutations in red lead to increased 4R tau relative to 3R tau. The asterisk near S352L denotes a single example of a lethal recessive tau mutation, which presented as respiratory hypoventilation and displayed neuronal tau pathology [85]. (b) Other mutations are found in the intron following exon 10, and these disrupt a hairpin structure in the pre-mRNA that regulates exon 10 inclusion/exclusion.

To date nearly 40 mutations associated with FTDP-17T or related disorders have been identified (reviewed in [124]) (Figure 4). These are all either missense mutations, mutations that affect splicing, or both, and almost all cluster in the portion encoding the C-terminal region or in an intervening sequence near exon 10. The C-terminal missense mutations all appear to impair tau binding to microtubules and the ability of tau to promote microtubule assembly, while a disease-associated mutation of a conserved arginine in the N-terminal region has been found to disrupt binding to the p150 subunit of the dynactin complex [125]. Most of the silent mutations increase the 4R-to-3R ratio by modulating alternative splicing of exon 10. Missense mutations found within exon 10 only affect the three 4R isoforms, while those found outside this region affect all six tau isoforms. Along with the consistent tau deposition in these familial cases and the observation that complete knockout of tau in mice does not lead to neurodegeneration, the fact that all but one of the disease-associated mutations [85] are dominant strongly suggests a gain of a toxic function: one normal copy remains, and in the case of exon 10 missense mutations, even the 3R tau translated from the disease allele is normal. No disease-associated mutations have been identified that lead to either a truncated protein or the nonsense-mediated decay of message.

Differences in the clinical and pathological phenotypes are seen between the various tau mutations (reviewed in [8, 124]). While many mutations lead to a phenotype resembling FTDP-17T, others lead to phenotypes overlapping or identical to Pick's disease, corticobasal degeneration, progressive supranuclear palsy, or AD. Some mutations lead to tau pathology in both neurons and glial cells, while others lead to tau pathology primarily or strictly in neurons. However, rather than suggesting specific phenotypic differences between tau mutations, many of these differences may reflect the different genetic and environmental contexts in which these mutations happen to reside. Ultrastructural differences in tau filaments in brain tissue are also observed between different tau mutations, with some correlating with twisted helical filaments, some with paired helical filaments (similar to what is seen in AD) and still others with straight filaments [124]. How these mutations may lead to differently assembled tau filaments is unclear.

#### 3.2.2. Effects of Mutations on Protein Aggregation

In general, missense mutations apparently affect tau's role in microtubule assembly. These mutations are primarily found in and around the microtubule binding domains and reduce the ability of tau to promote microtubule assembly from tubulin [126, 127]. Two exceptions are S305N and Q336R, which enhance the ability of tau to facilitate microtubule assembly [128, 129]. The S305N mutation alters exon 10 splicing [130]; thus, distinguishing the mechanism by which this mutation exerts pathogenicity is difficult. However, the ability of the Q336R mutation to enhance the microtubule binding role of tau again suggests that the balance of tau proteins capable of interacting with microtubules may be a critical factor ultimately dictating whether tau will self-assemble into filaments or not. Other evidence suggests that missense mutations may directly confer the ability of tau to form filaments. Some studies, for instance, show that a wide range of missense mutants (R5L, K257T, I260V, G272V, ΔK280, P301L, P301S, G335V, Q336R, V337 M, and R406W) promote *in vitro* formation of filaments in the presence of polymerization-inducing agents such as heparin and arachidonic acid [129, 131–137]. Filament formation has also been observed in neuroglioma cells upon expression of mutant tau [138]. 

Phosphorylation is also thought to be critical to the pathogenicity of tau (reviewed in [49]). Tau is phosphorylated in multiple sites by multiple kinases, but some sites tend to be more phosphorylated in tau pathology. Studying the role of these phosphorylations in tauopathies is fraught with difficulties however. *In vitro* phosphorylations by particular kinases are typically challenging to confirm *in vivo*, and the order of events (phosphorylation vis-à-vis microtubule dissociation or filament formation) is still unclear. Some kinase inhibitors have been shown to dramatically reduce tau pathology in transgenic mice [139, 140], but such agents notoriously lack specificity. Nevertheless, some evidence has been reported that disease-associated tau missense mutations can lead to enhanced phosphorylation [141], and hyperphosphorylation can induce tau self-assembly into filaments [142]. Other studies show that tau mutants bind less to phosphatase 2A [143], the principal phosphatase in the brain, which associates with the microtubule binding repeat domains.

#### 3.2.3. Effects of Mutations on Splicing

Roughly half of the identified tau mutations associated with FTDP-17 affect the alternative splicing of exon 10 (reviewed in [8, 124]) (Figure 4). While a number of these are intronic mutations near the exon 10 5′ splice site, others are mutations in the coding region of exon 10. Virtually all of these mutations lead to an increased inclusion of exon 10 and therefore an increase in the 4R-to-3R ratio.

The silent and intronic mutations that increase in 4R tau would be expected to have the opposite effect on microtubule binding to most of the missense mutations: the former would lead to increased binding while the latter decrease binding. How then can both types of mutations lead to filaments of hyperphosphorylated tau? One idea is that the increase in 4R tau saturates the binding sites on microtubules and thereby results in unassociated 4R tau that is more prone to phosphorylation and self-assembly [124]. Another possibility is that higher association with microtubules may create higher local concentrations of 4R tau and therefore a better microenvironment for assembly.

The silent and intronic mutations near the exon 10 5′ splice site enhance exon 10 inclusion through two general mechanisms: altering linear cis splicing elements or destabilizing a stem loop structure at the exon-intron junction. The stem loop structure was hypothesized [121, 122, 144] upon the initial discovery of FTDP-17 mutations in the tau gene, noting the apparent self-complementarity in this region. Subsequent determination of the solution structure of an oligonucleotide based on this exon-intron junction by nuclear magnetic resonance spectroscopy [145] led to refinement of the stem loop model to seven specific base pairs (Figure 4), with an adenosine bulge between the sixth and seventh base pair. The structure further shows that this unpaired purine ring is intercalated back into the A-form RNA duplex. Disease-associated mutations would be predicted to destabilize the stem loop and make this site more available to splicing factors (specifically the U1 snRNP that interacts with 5′ splice sites; see Figure 4).

Thermal stability studies of oligonucleotides demonstrated that disease-associated mutations within the putative stem loop lower the melting temperature of the RNA duplex (i.e., where the double-stranded RNA dissociates to the single-stranded form) [145, 146]. A minigene construct encoding exon 9–11 recapitulates normal tau exon 10 splicing for the wild-type sequence and increased exon 10 inclusion for disease-causing mutations [147]. This minigene has been used to demonstrate that other mutations specifically designed to enhance stability of the stem loop (and located distal to the U1 snRNP binding site) reduce exon 10 inclusion to decrease 4R/3R as predicted [146].

Other evidence has been taken to suggest that the stem loop is not a biologically relevant structure and that these intronic mutations instead either enhance the complementarity of the 5′ splice site with the U1 SNP or affect an intron splicing silencer (ISS) and the binding of a repressor protein [130, 148, 149]. However, U1 SNP complementarity changes cannot explain the effects of all the mutations in this region, an ISS cannot explain the effects of stem-loop stabilizing mutations, and no protein factor involved in ISS recognition at this site has been identified.

Other disease-causing mutations outside the stem loop can also increase exon 10 splicing, likely due to strengthening of an exon splicing enhancer (ESE) or weakening of an ISS or an exon splicing silencer (ESS). Putative ESE regions have been identified in exon 10 [148, 150], with one being purine-rich ESE and where mutations N279 K and ΔK280 reside. The splicing factor Tra2*β*, part of a class of RNA-binding proteins that contain serine- and arginine-rich (SR) domains, binds to this purine-rich ESE to enhance exon 10 inclusion [150]. In addition, an intron silencer modulator sequence element has been identified just downstream of the stem loop [149].

#### 3.2.4. Parkinson's and Others

Tau pathology and tau mutations are found in certain forms of parkinsonism, which is a PD-like movement disorder without all the defining pathological features of PD *per se* [151]. One example is FTDP-17T, mentioned earlier. However, recent genomewide association studies (GWAS) have identified the *MAPT* gene as a risk factor for sporadic PD [152–155]. This is especially surprising as PD typically does not display NFT pathology. In contrast, *MAPT* has not been identified as a genetic risk factor for AD, although AD is defined by the presence of tau pathology. Perhaps this is yet another piece of evidence that NFTs are not the form of tau that is most pathogenic. Interestingly, tau exon 10 splicing has been reported to be altered in PD, with an increase in 4R isoforms [156], similar to that observed with dominant FTD-associated MAPT mutations.

Tau pathology is also observed in chronic traumatic encephalopathy (CTE), in which multiple concussions or other head injuries elicit neurodegeneration with dementia and PD-like symptoms [157]. The disease is commonly found in athletes involved in contact sports and in military personnel exposed to blasts. One subtype of CTE is *dementia pugilistica*, named for its association with professional boxers, among whom Muhammad Ali is perhaps the most well-known case. The role of NFTs in pathogenesis is unclear in CTE, as it is in other tauopathies. However, the presence of similar pathology to that seen with tau mutations that cause other neurodegenerative diseases suggests that some form or forms of aberrant tau are responsible.

#### 3.2.5. Animal Models

A number of transgenic mice expressing tau have been developed in order to generate disease models for tauopathies, and some show tau pathology and neurodegeneration as well as behavioral deficits (reviewed in [9]). These mice have provided critical *in vivo* models for determining the role of aberrant tau in neurogeneration. For instance, evidence suggests that microgliosis and synaptic pathology may be the earliest manifestation of neurodegenerative tauopathies and that abrogation of tau-induce microglial activation may be therapeutically beneficial [96]. Crossing these mice with those that overproduce A*β* has provided important models for AD that have shed light on the role of tau with respect to A*β* [72]. A mouse line transgenic for human genomic *MAPT* expresses all six brain isoforms of tau and allows the study of the splicing of human tau *in vivo* [158]. Another mouse model likewise provides alternative splicing of the human transgene [159]. Moreover, knockout of endogenous mouse tau alleviates A*β*-induced memory deficits [47], suggesting that targeting tau may be a worthwhile AD therapeutic strategy, although another report suggests the opposite [70]. 

Questions remain regarding the pathological role of tau filaments and neurodegeneration. While most transgenic tau mice suggest that filament formation is connected to neurodegeneration (see [160]), results with a mouse line containing an inducible tau gene showed that suppressing tau improved memory even though neurofibrillary tangles continued to grow [99]. One important caveat to this study: tau levels under suppressed conditions in these mice were still dramatically higher than wild-type mice. Other evidence in worms, flies, and zebrafish suggest that tau can cause neurodegeneration in the absence of filament formation [97, 98, 161]. Nevertheless, it would be surprising if tau filaments within neuronal cell bodies and along axonal and dendritic projections are compatible with normal neuronal function and health. 

## 4. Strategies for Targeting Tau

### 4.1. Tau Kinases

As hyperphosphorylated tau is associated with NFTs and tau-related neurodegenerative diseases, inhibition of the responsible kinases has been a top strategy for targeting tau for the prevention or treatment of AD and other tauopathies [162]. Inhibitors of GSK3*β* and CDK5 have been of particular interest. Treatment with the GSK3 inhibitor LiCl induces a reduction of tau phosphorylation at Ser-202 and Ser-396/404 but not at Ser-262 or Ser-422 and has been shown to decrease tau aggregation and reduce axonal degeneration in tau transgenic mice [139].

LiCl is not selective for GSK3*β* versus GSK3*α* and can have many other off-target effects, making assessment of its ability to inhibit tau phosphorylation via GSK3*β* problematic. However, an apparently selective GSK3 inhibitor AR-A014418 was also found to reduce tau phosphorylation, filamentous tau, and axonal degeneration after oral administration for one month in tau transgenic mice [139]. An analog of AR-A014418 entered into clinical trials for AD in 2007, but its development has since been discontinued. Another selective GSK3 inhibitor, Nypta (Tideglusib), is noncompetitive for ATP [163] and appears to be well tolerated and to improve cognitive performance in a 4–6-week clinical trial [164]. Although the study was not large enough to be statistically significant and target engagement was not clear, Tideglusib has entered Phase IIb trials.

While such results seem promising in many ways, GSK3 is important for normal human physiology; as its name implies, it is critical to proper glycogen metabolism. Moreover, a selective GSK3 inhibitor was found to cause neurodegeneration in healthy wild-type mice [165], suggesting that at least some level of GSK3 activity is essential to neuronal function. Therefore, although abnormally high GSK3 activity is associated with several different diseases (diabetes, cancer, and bipolar disorder) and GSK3 appears to be a promising target, complete inhibition is apparently not desirable.

Cdk5 has also been considered a potentially worthwhile target, especially as this kinase is not involved in the cell cycle and is active in postmitotic cells [166]. However, the development of specific Cdk5 inhibitors has proven challenging. Other cdks as well as GSK3 are typically affected as well. Dual Cdk5/GSK3 inhibitors may be desirable, but other cdks should be avoided so as not to interfere with cell replication. Relatively nonspecific kinase inhibitors have also been considered, with the idea that multiple kinases phosphorylate tau, and hitting many of these partially would reduce tau phosphorylation while minimizing toxicity. Indeed, one such compound, SRN-003-556, which inhibits GSK3, cdk1, PKA, PKC, and ERK2 to similar degrees, reduced tau phosphorylation and reduced motor deficits in a tau transgenic mouse model but without affecting NFT count [140], further evidence that NFTs *per se* may not be pathogenic. Nevertheless, great care should be taken to define the range of targets of this compound and to profile its toxicology.

### 4.2. Tau Aggregation

The assembly of tau into neurotoxic oligomers or fibrils makes blocking tau-tau interactions a reasonable strategy for therapeutic intervention. A number of screens in search of compounds that inhibit tau self-assembly have been carried out, and a variety of compound types have been identified, including phenothiazines, porphyrins, polyphenols, cyanine dyes, aminothienopyrazines, anthraquinones, phenothiazolyl hydrazides, and rhodanines [167]. Many of these screens were carried out using the binding of thioflavin T as a readout. Thioflavin T binds to cross-*β*-fibril structures of many assemble proteins, including A*β* and tau, and such binding results in fluorescence. Compounds identified in such screens may inhibit fibril formation but not soluble oligomers and other assemblies. Indeed, the potential to increase soluble oligomers exists. Therefore, counterscreens are needed to ensure that compounds block early steps in the aggregation process. One example is to tag tau with a fluorescent moiety and use fluorescence polarization as a readout, as increased size of the assemblies (e.g., monomer to dimer to trimer, etc.) results in an increased signal [168].

The most advanced tau assembly inhibitor is the phenothiazine dye methylene blue (Rember). In 2008, this compound was reported to slow cognitive decline in a small Phase II clinical trial over almost one year, although this result was complicated by the fact that the highest dose led to lower than expected drug exposures, and no evidence for target engagement was reported [169]. A study in a tau transgenic mouse model, however, showed that very high doses that lowered soluble tau levels also resulted in cognitive improvement [170]. While a Phase III trial would help clarify the efficacy of methylene blue in humans, no such trial has yet been initiated. However, a second-generation methylene blue analog, LMTX, is allegedly in line for a Phase III trial. No other tau aggregation inhibitors are in clinical trials at this time.

Among the more promising new approaches to the search for effective tau assembly inhibitors is a structure-based design strategy, targeting the cross-*β*-sheet structure of aggregated tau. While the detailed structure of tau fibrils is unknown, the structure of fibrils of the tau peptide VQIVYK, residues 306–311, was deduced by x-ray microcrystallography [171] and found to be a “steric zipper” of *β*-sheets. This short tau peptide is critical for tau assembly in the context of the full-length protein and on its own assemble into fibrils similar to that formed by tau itself. D-peptides were computationally designed to strongly interact with this structure and prevent fibril elongation [172]. One such peptide was found to delay fibril formation, even at substoichiometric concentrations, and could also inhibit fibril elongation. Small changes to the structure of this D-peptide rendered it ineffective, and the active peptide was found to have no effect on A*β* fibril formation, a remarkable demonstration of specificity. While this D-peptide itself is not a good drug candidate (peptides, even D-peptides, rarely are), it may be a reasonable starting point for drug design and validates the structure-based design approach.

Despite some promising advances in tau aggregation inhibitors, a number of issues will need to be addressed to facilitate translation into the clinic. First, the stoichiometry required to effectively engage tau and show efficacy should be understood for a given agent. In most cases, tau aggregation inhibitors need to be at least equimolar with tau protein in order to have the desired effects. Secondly, the specificity of the compounds must be established to ensure that normal protein-protein interactions are not unduly affected. Third, the steps in tau assembly that are affected by the compound should be elucidated. As mentioned earlier, fibrils *per se* may not be the primary neurotoxic entity, and blocking fibril formation alone may lead to the buildup of more deleterious soluble oligomeric assemblies. Fourth, the ability of the compounds to interact with free tau versus microtubule-bound tau should be determined or otherwise address whether there is interference with normal tau functions. Although the knockout of tau in mice leads to viable, fertile, and (for the most part) healthy mice, the effect of conditionally knocking out tau is presently unknown.

### 4.3. Tau Clearance

Stimulating the natural ability of cells to clear away misfolded or aggregated proteins has been another strategy for targeting aberrant tau. One specific approach is by enhancing degradation of tau by the ubiquitin-proteasome system. Misfolded proteins are tagged with chains of ubiquitin that enable interaction with and breakdown by the proteasome. This process can apparently be stimulated with inhibitors of the ATPase activity of heat shock protein (HSP) 90, a molecular chaperone involved in protein refolding. Inhibition of this HSP90 activity results in increased expression of HSP70, which together with CHIP (the carboxyterminus of HSP70-interacting protein) is involved in the ubiquitination of misfolded proteins [173]. Knockout of CHIP in mice results in elevated phosphorylated tau, suggesting that HSP70/CHIP is involved in the degradation of this particular form of tau [174]. Consistent with this notion, HSP90 inhibitors increase proteasomal degradation of phosphorylated tau through HSP70/CHIP in cell culture [175, 176] and decrease hyperphosphorylated tau in tau transgenic mice [176, 177].

Toward the identification of small-molecule stimulators of the ubiquitin-proteasome system to enhance the clearance of deleterious proteins, the discovery that a proteasome-associated deubiquitinating enzyme, USP14, inhibits proteasomal degradation of proteins by trimming off ubiquitin chains inspired a high-throughput screen to find inhibitors of USP14 activity [120]. The hit compound that emerged from this screen, dubbed IU1, reversibly inhibited USP14 and did so selectively over other deubiquitinating enzymes. IU1 could also stimulate the degradation of tau in a concentration-dependent manner in cell culture, and this tau degradation required USP14 and active proteasome complexes. The degradation of another protein implicated in neurodegeneration, TDP-43, was likewise enhanced by IU1, while a ubiquitin-independent substrate of the proteasome was not affected.

Monomeric, misfolded tau can be degraded by the proteasome, but aggregated forms of tau cannot, as such aggregates cannot pass through the proteasome pore. However, protein aggregates as well as whole intracellular organelles can be cleared through a process called autophagy (literally, self-eating) [178]. In one type of autophagy (macroautophagy) protein aggregates or organelles are enveloped into vesicles that then fuse with lysosomes for degradation. Some evidence suggests that reduction of this process can lead to neurodegeneration [179, 180] and that enhancement of autophagy can help clear out aggregated proteins associated with neurodegeneration [181]. The immunosuppressant drug rapamycin can enhance macroautophagy and prevent plaques, NFTs, and cognitive deficits in an AD mouse model [182]. However, the safety of this agent for long-term treatment of AD in humans is a serious concern. Recent results suggest that trehalose also activates macroautophagy, reduces insoluble tau, and is neuroprotective in a tau transgenic mouse model [183], although trehalose has multiple biological effects, and the safety of this agent at chronic pharmacological doses is likewise unclear. Other means of pharmacologically increasing autophagy that are more specific may be required, but even then, the consequences of chronic stimulation of autophagy are unclear.

### 4.4. Antibodies

Active and passive immunization targeting tau is another promising approach for the prevention and treatment of AD and other tauopathies [184]. The impetus for this approach was promising findings on the use of anti-A*β* antibodies in transgenic mouse models, demonstrating that A*β* plaques could be prevented in younger mice and even cleared in older mice and that cognitive deficiencies in these mice could be prevented or reversed, even upon peripheral administration. These results have led to a number of clinical trials for the treatment of AD. Despite the preconceived notion that antibodies cannot cross the blood-brain barrier, studies show that low levels of anti-A*β* antibodies do access the brain and elicit clearance of amyloid plaques. However, these plaques are extracellular, while tau and tau pathology is largely intraneuronal, suggesting that anti-tau antibodies may not be capable of engaging their target. Nevertheless, initial evidence shows that tau pathology can be reduced and behavioural phenotypes slowed by active immunization with antiphosphotau peptide in tau transgenic mice [185–188]. Passive immunization has likewise shown promise [189, 190].

How might antibodies prevent tau pathology? One possibility is that they enter neurons through endocytosis, although there is no evidence that this occurs, and even if it did, the antibodies would have to somehow escape the endocytic vesicles to gain access to tau in the cytoplasm. Another possibility is that tau is secreted from neurons to some degree and that this allows the spread of tau pathology from neuron to neuron, a mechanism discussed earlier. In this scenario, anti-tau antibodies would interact with tau at synapses and intercept misfolded or aggregated tau before it is taken up by the neighboring neuron. Consistent with this idea is the fact that tau is found in the CSF and is considered a promising biomarker for AD [88].

### 4.5. Tau RNA

Because of the difficulty in targeting the tau protein directly (it is not obviously “druggable,” as it has no natural “ligands” *per se*) and the uncertainty of targeting enzymes that modulate tau (e.g., kinases), some have considered targeting the tau mRNA to either lower overall tau protein levels or to shift splicing in a potentially therapeutically productive way. The idea of knocking down the tau message with siRNA or reducing tau protein translation via antisense oligonucleotides is based on the findings in mice that knockout of the *MAPT* gene attenuates the learning and memory deficits of FAD-mutant APP/presenilin transgenic mice [47], which suggests that tau is critical to the pathogenic process initiated by aggregated forms of A*β*. Although most reports on *MAPT* knockout suggest that the mice are normal, fertile, and healthy, the possibility of compensation (e.g., by MAP1) cannot be excluded. As no conditional *MAPT* knockout mice have yet been reported, it is unclear if tau is critical for normal neuronal function *in vivo*, so complete ablation of tau by siRNA or antisense oligonucleotides may not be desirable. Also, another report describes crossing a tau knockout with a human mutant APP mouse leading to increased axonal degeneration [70].

Shifting the splicing of tau pre-mRNA is a more subtle strategy that should not affect overall levels of tau protein. The rationale for this approach is the finding that nearly half of the ~40 mutations in the *MAPT* gene that are associated with FTD alter the balance of tau exon 10 splicing to increase the ratio or 4R to 3R tau isoforms. This skewing of tau splicing, with no change in the encoded wild-type protein sequence, is sufficient for tau pathology, neurodegeneration, and dementia. Therefore, use of agents that can shift the splicing back toward a more normal 1 : 1 physiological ratio, (or even in favor of 3R) may be beneficial in treating these FTDs. Such agents might also be therapeutic for AD as well, although there is little evidence that the 4R-to-3R ratio is altered in AD. Antisense oligonucleotides that target the exon10-intron10 boundary can shift splicing in this potentially beneficial way. These include antisense directed to the linear sequence at this boundary [191] as well as bipartite oligonucleotides [192] directed to the 5′ and 3′ regions that flank the hairpin structure at this boundary, which prevent access by the U1 snRNP. The search for small, drug-like molecules that bind and stabilize this hairpin has also identified compounds such as neomycin and mitoxantrone [193–197]. However, these compounds lack specificity for the tau RNA hairpin structure.

### 4.6. Other Strategies

Several other approaches to targeting tau or compensating for potential loss of tau function have been explored. One of these is to target the prolylisomerase PIN1, which switches specific tau p-Ser/Thr-Pro motifs between trans- and cis-rotamer forms [198]. Recent evidence using tau pT231-P232 rotamer-specific antibodies [199] suggests that the cis-, but not the trans-, rotamer is an early conformer associated with AD progression; that PIN1 restores the ability of cis-pT231-Tau to promote microtubule assemble; that cis-pT231-Tau is more stable and more prone to aggregation than trans-pT231-Tau; that cis-, but not trans-, pT231-Tau correlates with NFTs in the AD hippocampus; that PIN1 promotes cis-to-trans isomerization of pT231-Tau in AD mouse models. Thus, increasing PIN1 activity may be a worthwhile strategy for therapeutic intervention for AD at the level of tau. How to do this, however, is not obvious. Alternatively, immunotherapy with the conformer-specific antibodies might block the pathogenic functions of cis-pT231-Tau.

Reducing the acetylation of tau may be another reasonable approach for lowering pathogenic forms of tau [200]. Acetylation at lysines often competes with ubiquitination at the same sites. Thus, acetylation can prevent the degradation of misfolded tau by the ubiquitin-proteasome system. Tau is acetylated by the lysine acetyltransferase p300 and deacetylated by the lysine deacetylase SIRT1 [201]. Thus, inhibition of p300 activity or stimulation of SIRT1 activity might facilitate ubiquitination and proteasomal degradation of misfolded tau. Indeed, a small molecule inhibitor of p300 has been shown to prevent neuronal accumulation of phosphorylated tau [201]. However, p300 and SIRT1 have many substrates, raising questions about the consequences of altering their levels of activity.

Another approach is to pharmacologically rescue the potential loss of function of tau in stabilizing microtubules. While the knockout of tau does not result in neurodegeneration and none of the FTD-associated MAPT mutations result in truncation or deletion of large regions of the protein, it remains possible that misfolded or aggregated tau might lead to insufficient levels of tau protein for microtubule stabilization. For this reason, antimitotic agents that act as microtubule stabilizers have been explored for their ability to rescue this tau function. One such agent, paclitaxel, prevented axonal transport deficits and motor impairments in a tau transgenic mouse [202], and epothilone D improved microtubule density, axonal integrity, and cognition of another tau mouse model [203], even in aged mice that already had pathology [204]. An epothilone analog has likewise shown benefit [205]. A peptide called NAP also stabilizes microtubules and (surprisingly) reduces tau hyperphosphorylation and ameliorates cognitive deficits in tau mouse models [206–208]. Intranasal delivery of NAP has shown some promise in a phase II clinical trial.

## 5. Conclusions and Perspective

Substantial evidence supports the tau protein as a key contributor to the pathogenesis of AD. However, the form of tau that is neurotoxic, the means by which this toxicity is transmitted, and the role of A*β* in triggering aberrant tau are all unresolved issues. Misfolded and/or aggregated tau protein appears to be responsible, but the exact nature of the pathogenic form(s) (e.g., oligomeric state and degree of phosphorylation) remains unclear. Despite this uncertainty, genetic studies in mice suggest that reducing tau expression should be therapeutic for AD, and FTD-causing mutations in the *MAPT* gene itself indicate a pathogenic role of aberrant tau. Moreover, as tau pathology correlates better with neurodegeneration than amyloid plaques, pharmacological intervention at the tau level may be efficacious later in the disease process than it would be at the A*β* level, which may require presymptomatic diagnosis and treatment.

**Figure 5:**
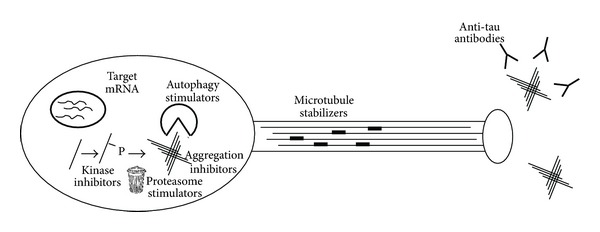
Targets for pharmacological modulation of tau and tau function. See text for details.

Many different strategies have been pursued in the hopes of intervening therapeutically at the level of tau (Figure 5). Nevertheless, tau remains relatively underdeveloped as a therapeutic target, especially compared with A*β*. This is due to the unresolved issues mentioned previously along with the fact that the tau protein itself is not a typical “druggable” target, with endogenous small molecule ligands or otherwise containing pockets to which drug molecules might obviously bind in a pharmacologically meaningful way. Together, these challenges make it difficult to decide on a suitable strategy for drug discovery. More basic research is needed to understand the role of tau in neurodegenerative diseases to identify new targets and approaches for pharmacological intervention. In addition, the delivery of tau-targeting agents to the brain is a major impediment; novel delivery approaches to the brain, such as nanoparticles [209], should be explored in conjunction with drug discovery.

In the meantime, however, one or more of the current approaches may eventually bear fruit in the form of an approved drug for AD and/or related disorders, so these efforts should continue. The medical need is simply too great to halt the ongoing search for an effective means of targeting tau. Only one or a few of the many reasonable approaches need to effectively prevent or delay the onset or progression of tauopathies, and at this point it is impossible to say which of these approaches might be successful. Thus, all reasonable strategies should be pursued in the hope of finding disease-modifying therapies for these devastating brain disorders.

## References

[1] Powers ET, Morimoto RI, Dillin A, Kelly JW, Balch WE (2009). Biological and chemical approaches to diseases of proteostasis deficiency. *Annual Review of Biochemistry*.

[2] Hartl FU, Bracher A, Hayer-Hartl M (2011). Molecular chaperones in protein folding and proteostasis. *Nature*.

[3] Stamatoyannopoulos G (1972). The molecular basis of hemoglobin disease. *Annual Review of Genetics*.

[4] Plante-Bordeneuve V, Said G (2011). Familial amyloid polyneuropathy. *Lancet Neurology*.

[5] Ross CA, Poirier MA (2004). Protein aggregation and neurodegenerative disease. *Nature Medicine*.

[6] Kuemmerle S, Gutekunst CA, Klein AM (1999). Huntington aggregates may not predict neuronal death in Huntington's disease. *Annals of Neurology*.

[7] Chesebro B, Trifilo M, Race R (2005). Medicine: anchorless prion protein results in infectious amyloid disease without clinical scrapie. *Science*.

[8] Lee VMY, Goedert M, Trojanowski JQ (2001). Neurodegenerative tauopathies. *Annual Review of Neuroscience*.

[9] Ballatore C, Lee VMY, Trojanowski JQ (2007). Tau-mediated neurodegeneration in Alzheimer’s disease and related disorders. *Nature Reviews Neuroscience*.

[10] Goedert M, Spillantini MG (2006). A century of Alzheimer’s disease. *Science*.

[11] Wolfe MS (2009). Tau mutations in neurodegenerative diseases. *Journal of Biological Chemistry*.

[12] Braak H, Braak E (1991). Neuropathological stageing of Alzheimer-related changes. *Acta Neuropathologica*.

[13] Gomez-Isla T (1996). Profound loss of layer II entorhinal cortex neurons occurs in very mild Alzheimer's disease. *Journal of Neuroscience*.

[14] Hyman BT, Van Hoesen GW, Damasio AR, Barnes CL (1984). Alzheimer’s disease: cell-specific pathology isolates the hippocampal formation. *Science*.

[15] de Calignon A, Polydoro M, Suarez-Calvet M (2012). Propagation of tau pathology in a model of early Alzheimer's disease. *Neuron*.

[16] Liu L, Drouet V, Wu JW (2012). Trans-synaptic spread of tau pathology in vivo. *PLoS One*.

[17] Pittman AM, Fung HC, de Silva R (2006). Untangling the tau gene association with neurodegenerative disorders. *Human Molecular Genetics*.

[18] Hardy J, Pittman A, Myers A (2005). Evidence suggesting that Homo neanderthalensis contributed the H2 MAPT haplotype to Homo sapiens. *Biochemical Society Transactions*.

[19] Green RE (2010). A draft sequence of the Neandertal genome. *Science*.

[20] Baker M, Litvan I, Houlden H (1999). Association of an extended haplotype in the tau gene with progressive supranuclear palsy. *Human Molecular Genetics*.

[21] Healy DG, Abou-Sleiman PM, Lees AJ (2004). Tau gene and Parkinson’s disease: a case-control study and meta-analysis. *Journal of Neurology, Neurosurgery and Psychiatry*.

[22] Pittman AM, Myers AJ, Abou-Sleiman P (2005). Linkage disequilibrium fine mapping and haplotype association analysis of the tau gene in progressive supranuclear palsy and corticobasal degeneration. *Journal of Medical Genetics*.

[23] Myers AJ, Kaleem M, Marlowe L (2005). The H1c haplotype at the MAPT locus is associated with Alzheimer’s disease. *Human Molecular Genetics*.

[24] Caffrey TM, Joachim C, Paracchini S, Esiri MM, Wade-Martins R (2006). Haplotype-specific expression of exon 10 at the human MAPT locus. *Human Molecular Genetics*.

[25] Myers AJ, Pittman AM, Zhao AS (2007). The MAPT H1c risk haplotype is associated with increased expression of tau and especially of 4 repeat containing transcripts. *Neurobiology of Disease*.

[26] Gu Y, Oyama F, Ihara Y (1996). *τ* Is widely expressed in rat tissues. *Journal of Neurochemistry*.

[27] Weingarten MD, Lockwood AH, Hwo SY, Kirschner MW (1975). A protein factor essential for microtubule assembly. *Proceedings of the National Academy of Sciences of the United States of America*.

[28] Cleveland DW, Hwo SY, Kirschner MW (1977). Purification of tau, a microtubule associated protein that induces assembly of microtubules from purified tubulin. *Journal of Molecular Biology*.

[29] Binder LI, Frankfurter A, Rebhun LI (1985). The distribution of tau in the mammalian central nervous system. *Journal of Cell Biology*.

[30] Ittner LM, Ke YD, Delerue F (2010). Dendritic function of tau mediates amyloid-*β* toxicity in alzheimer’s disease mouse models. *Cell*.

[31] Kowall NW, Kosik KS (1987). Axonal disruption and aberrant localization of tau protein characterize the neuropil pathology of Alzheimer’s disease. *Annals of Neurology*.

[32] Himmler A, Drechsel D, Kirschner MW, Martin DW (1989). Tau consists of a set of proteins with repeated C-terminal microtubule-binding domains and variable N-terminal domains. *Molecular and Cellular Biology*.

[33] Lee G, Neve RL, Kosik KS (1989). The microtubule binding domain of tau protein. *Neuron*.

[34] Butner KA, Kirschner MW (1991). Tau protein binds to microtubules through a flexible array of distributed weak sites. *Journal of Cell Biology*.

[35] Gustke N, Trinczek B, Biernat J, Mandelkow EM, Mandelkow E (1994). Domains of *τ* protein and interactions with microtubules. *Biochemistry*.

[36] Andreadis A, Brown WM, Kosik KS (1992). Structure and novel exons of the human *τ* gene. *Biochemistry*.

[37] Goedert M, Spillantini MG, Jakes R, Rutherford D, Crowther RA (1989). Multiple isoforms of human microtubule-associated protein tau: sequences and localization in neurofibrillary tangles of Alzheimer’s disease. *Neuron*.

[38] Goedert M, Spillantini MG, Potier MC, Ulrich J, Crowther RA (1989). Cloning and sequencing of the cDNA encoding an isoform of microtubule-associated protein tau containing four tandem repeats: differential expression of tau protein mRNAs in human brain. *EMBO Journal*.

[39] Wang J, Tse SW, Andreadis A (2007). Tau exon 6 is regulated by an intricate interplay of trans factors and cis elements, including multiple branch points. *Journal of Neurochemistry*.

[40] Goedert M, Jakes R (1990). Expression of separate isoforms of human tau protein: Correlation with the tau pattern in brain and effects on tubulin polymerization. *EMBO Journal*.

[41] Shaw-Smith C, Pittman AM, Willatt L (2006). Microdeletion encompassing MAPT at chromosome 17q21.3 is associated with developmental delay and learning disability. *Nature Genetics*.

[42] Koolen DA, Vissers LELM, Pfundt R (2006). A new chromosome 17q21.31 microdeletion syndrome associated with a common inversion polymorphism. *Nature Genetics*.

[43] Sapir T, Frotscher M, Levy T (2012). Tau's role in the developing brain: implications for intellectual disability. *Human Molecular Genetics*.

[44] Dawson HN, Ferreira A, Eyster MV, Ghoshal N, Binder LI, Vitek MP (2001). Inhibition of neuronal maturation in primary hippocampal neurons from tau deficient mice. *Journal of Cell Science*.

[45] Harada A, Oguchi K, Okabe S (1994). Altered microtubule organization in small-calibre axons of mice lacking tau protein. *Nature*.

[46] Ikegami S, Harada A, Hirokawa N (2000). Muscle weakness, hyperactivity, and impairment in fear conditioning in tau-deficient mice. *Neuroscience Letters*.

[47] Roberson ED, Scearce-Levie K, Palop JJ (2007). Reducing endogenous tau ameliorates amyloid *β*-induced deficits in an Alzheimer’s disease mouse model. *Science*.

[48] Yuan A, Kumar A, Peterhoff C, Duff K, Nixon RA (2008). Axonal transport rates in vivo are unaffected by tau deletion or overexpression in mice. *Journal of Neuroscience*.

[49] Johnson GVW, Stoothoff WH (2004). Tau phosphorylation in neuronal cell function and dysfunction. *Journal of Cell Science*.

[50] Drewes G, Trinczek B, Illenberger S (1995). Microtubule-associated protein/microtubule affinity-regulating kinase (p110(mark)). A novel protein kinase that regulates tau-microtubule interactions and dynamic instability by phosphorylation at the Alzheimer-specific site serine 262. *Journal of Biological Chemistry*.

[51] Drewes G, Ebneth A, Preuss U, Mandelkow EM, Mandelkow E (1997). MARK, a novel family of protein kinases that phosphorylate microtubule- associated proteins and trigger microtubule disruption. *Cell*.

[52] Biernat J, Wu YZ, Timm T (2002). Protein kinase MARK/PAR-1 is required for neurite outgrowth and establishment of neuronal polarity. *Molecular Biology of the Cell*.

[53] Mandelkow EM, Thies E, Trinczek B, Biernat J, Mandelkow E (2004). MARK/PAR1 kinase is a regulator of microtubule-dependent transport in axons. *Journal of Cell Biology*.

[54] Doble BW, Woodgett JR (2003). GSK-3: tricks of the trade for a multi-tasking kinase. *Journal of Cell Science*.

[55] Su SC, Tsai LH Cyclin-dependent kinases in brain development and disease. *Annual Review of Cell and Developmental Biology*.

[56] Fleming LM, Johnson GVW (1995). Modulation of the phosphorylation state of tau in situ: the roles of calcium and cyclic AMP. *Biochemical Journal*.

[57] Litersky JM, Johnson GVW, Jakes R, Goedert M, Lee M, Seubert P (1996). Tau protein is phosphorylated by cyclic AMP-dependent protein kinase and calcium/calmodulin-dependent protein kinase II within its microtubule-binding domains at Ser-262 and Ser-356. *Biochemical Journal*.

[58] Mandell JW, Banker GA (1996). A spatial gradient of tau protein phosphorylation in nascent axons. *Journal of Neuroscience*.

[59] Dixit R, Ross JL, Goldman YE, Holzbaur ELF (2008). Differential regulation of dynein and kinesin motor proteins by tau. *Science*.

[60] Ebneth A, Godemann R, Stamer K (1998). Overexpression of tau protein inhibits kinesin-dependent trafficking of vesicles, mitochondria, and endoplasmic reticulum: Implications for Alzheimer’s disease. *Journal of Cell Biology*.

[61] Qiang L, Yu W, Andreadis A, Luo M, Baas PW (2006). Tau protects microtubules in the axon from severing by katanin. *Journal of Neuroscience*.

[62] Takei Y, Teng J, Harada A, Hirokawa N (2000). Defects axonal elongation and neuronal migration in mice with disrupted tau and map1b genes. *Journal of Cell Biology*.

[63] Morris M, Maeda S, Vossel K, Mucke L (2011). The Many Faces of Tau. *Neuron*.

[64] Klein C, Kramer EM, Cardine AM, Schraven B, Brandt R, Trotter J (2002). Process outgrowth of oligodendrocytes is promoted by interaction of Fyn kinase with the cytoskeletal protein Tau. *Journal of Neuroscience*.

[65] Selkoe DJ (2001). Alzheimer’s disease: Genes, proteins, and therapy. *Physiological Reviews*.

[66] Cole SL, Vassar R (2008). The role of amyloid precursor protein processing by BACE1, the *β*-secretase, in Alzheimer disease pathophysiology. *Journal of Biological Chemistry*.

[67] Wolfe MS (2006). The *γ*-secretase complex: membrane-embedded proteolytic ensemble. *Biochemistry*.

[68] Tanzi RE, Bertram L (2005). Twenty years of the Alzheimer’s disease amyloid hypothesis: a genetic perspective. *Cell*.

[69] Ashe KH, Zahs KR (2010). Probing the biology of Alzheimer’s disease in mice. *Neuron*.

[70] Dawson HN, Cantillana V, Jansen M (2010). Loss of tau elicits axonal degeneration in a mouse model of Alzheimer’s disease. *Neuroscience*.

[71] Oddo S, Caccamo A, Shepherd JD (2003). Triple-transgenic model of Alzheimer’s Disease with plaques and tangles: Intracellular A*β* and synaptic dysfunction. *Neuron*.

[72] Oddo S, Billings L, Kesslak JP, Cribbs DH, LaFerla FM (2004). A*β* immunotherapy leads to clearance of early, but not late, hyperphosphorylated tau aggregates via the proteasome. *Neuron*.

[73] De Felice FG, Wu D, Lambert MP (2008). Alzheimer’s disease-type neuronal tau hyperphosphorylation induced by A*β* oligomers. *Neurobiology of Aging*.

[74] Zempel H, Thies E, Mandelkow E, Mandelkow EM (2010). A*β* oligomers cause localized Ca^2+^ elevation, missorting of endogenous Tau into dendrites, Tau phosphorylation, and destruction of microtubules and spines. *Journal of Neuroscience*.

[75] Jin M, Shepardson N, Yang T, Chen G, Walsh D, Selkoe DJ (2011). Soluble amyloid *β*-protein dimers isolated from Alzheimer cortex directly induce Tau hyperphosphorylation and neuritic degeneration. *Proceedings of the National Academy of Sciences of the United States of America*.

[76] Nussbaum JM (1038). Prion-like behaviour and tau-dependent cytotoxicity of pyroglutamylated amyloid-beta. *Nature*.

[77] Laurén J, Gimbel DA, Nygaard HB, Gilbert JW, Strittmatter SM (2009). Cellular prion protein mediates impairment of synaptic plasticity by amyloid-*β* oligomers. *Nature*.

[78] Chin J, Palop JJ, Yu GQ, Kojima N, Masliah E, Mucke L (2004). Fyn kinase modulates synaptotoxicity, but not aberrant sprouting, in human amyloid precursor protein transgenic mice. *Journal of Neuroscience*.

[79] Chin J, Palop JJ, Puoliväli J (2005). Fyn kinase induces synaptic and cognitive impairments in a transgenic mouse model of Alzheimer’s disease. *Journal of Neuroscience*.

[80] Roberson ED, Halabisky B, Yoo JW (2011). Amyloid-*β*/fyn-induced synaptic, network, and cognitive impairments depend on tau levels in multiple mouse models of alzheimer’s disease. *Journal of Neuroscience*.

[81] Frost B, Jacks RL, Diamond MI (2009). Propagation of Tau misfolding from the outside to the inside of a cell. *Journal of Biological Chemistry*.

[82] Guo JL, Lee VMY (2011). Seeding of normal tau by pathological tau conformers drives pathogenesis of Alzheimer-like tangles. *Journal of Biological Chemistry*.

[83] Nonaka T, Watanabe ST, Iwatsubo T, Hasegawa M (2010). Seeded aggregation and toxicity of *α*-synuclein and tau: Cellular models of neurodegenerative diseases. *Journal of Biological Chemistry*.

[84] Clavaguera F, Bolmont T, Crowther RA (2009). Transmission and spreading of tauopathy in transgenic mouse brain. *Nature Cell Biology*.

[85] Nicholl DJ, Greenstone MA, Clarke CE (2003). An English kindred with a novel recessive tauopathy and respiratory failure. *Annals of Neurology*.

[86] Caughey B, Baron GS (2006). Prions and their partners in crime. *Nature*.

[87] Saman S, Kim W, Raya M (2012). Exosome-associated tau is secreted in tauopathy models and is selectively phosphorylated in cerebrospinal fluid in early Alzheimer disease. *Journal of Biological Chemistry*.

[88] Blennow K, Hampel H, Weiner M, Zetterberg H (2010). Cerebrospinal fluid and plasma biomarkers in Alzheimer disease. *Nature Reviews Neurology*.

[89] Nelson PT, Alafuzoff I, Bigio EH (2012). Correlation of Alzheimer disease neuropathologic changes with cognitive status: a review of the literature. *Journal of Neuropathology & Experimental Neurology*.

[90] Haass C, Selkoe DJ (2007). Soluble protein oligomers in neurodegeneration: Lessons from the Alzheimer’s amyloid *β*-peptide. *Nature Reviews Molecular Cell Biology*.

[91] Maeda S, Sahara N, Saito Y, Murayama S, Ikai A, Takashima A (2006). Increased levels of granular tau oligomers: An early sign of brain aging and Alzheimer’s disease. *Neuroscience Research*.

[92] Peterson DW, Zhou H, Dahlquist FW, Lew J (2008). A soluble oligomer of tau associated with fiber formation analyzed by NMR. *Biochemistry*.

[93] Lasagna-Reeves CA, Castillo-Carranza DL, Sengupta U (2012). Identification of oligomers at early stages of tau aggregation in Alzheimer's disease. *The FASEB Journal*.

[94] Andorfer C, Acker CM, Kress Y, Hof PR, Duff K, Davies P (2005). Cell-cycle reentry and cell death in transgenic mice expressing nonmutant human tau isoforms. *Journal of Neuroscience*.

[95] Polydoro M, Acker CM, Duff K, Castillo PE, Davies P (2009). Age-dependent impairment of cognitive and synaptic function in the htau mouse model of Tau pathology. *Journal of Neuroscience*.

[96] Yoshiyama Y, Higuchi M, Zhang B (2007). Synapse loss and microglial activation precede tangles in a P301S tauopathy mouse model. *Neuron*.

[97] Wittmann CW, Wszolek MF, Shulman JM (2001). Tauopathy in drosophila: neurodegeneration without neurofibrillary tangles. *Science*.

[98] Kraemer BC, Zhang B, Leverenz JB, Thomas JH, Trojanowski JQ, Schellenberg GD (2003). Neurodegeneration and defective neurotransmission in a Caenorhabditis elegans model of tauopathy. *Proceedings of the National Academy of Sciences of the United States of America*.

[99] Santacruz K, Lewis J, Spires T (2005). Medicine: Tau suppression in a neurodegenerative mouse model improves memory function. *Science*.

[100] Berger Z, Roder H, Hanna A (2007). Accumulation of pathological tau species and memory loss in a conditional model of tauopathy. *Journal of Neuroscience*.

[101] Spires TL, Orne JD, SantaCruz K (2006). Region-specific dissociation of neuronal loss and neurofibrillary pathology in a mouse model of tauopathy. *American Journal of Pathology*.

[102] Maeda S, Sahara N, Saito Y (2007). Granular tau oligomers as intermediates of tau filaments. *Biochemistry*.

[103] Bancher C, Brunner C, Lassmann H (1989). Accumulation of abnormally phosphorylated *τ* precedes the formation of neurofibrillary tangles in Alzheimer’s disease. *Brain Research*.

[104] Wang Y, Garg S, Mandelkow EM, Mandelkow E (2010). Proteolytic processing of tau. *Biochemical Society Transactions*.

[105] Rissman RA, Poon WW, Blurton-Jones M (2004). Caspase-cleavage of tau is an early event in Alzheimer disease tangle pathology. *Journal of Clinical Investigation*.

[106] Guillozet-Bongaarts AL, Cahill ME, Cryns VL, Reynolds MR, Berry RW, Binder LI (2006). Pseudophosphorylation of tau at serine 422 inhibits caspase cleavage: in vitro evidence and implications for tangle formation in vivo. *Journal of Neurochemistry*.

[107] Delobel P, Lavenir I, Fraser G (2008). Analysis of tau phosphorylation and truncation in a mouse model of human tauopathy. *American Journal of Pathology*.

[108] Zhang Q, Zhang X, Chen J, Miao Y, Sun A (2009). Role of caspase-3 in tau truncation at D421 is restricted in transgenic mouse models for tauopathies. *Journal of Neurochemistry*.

[109] Lerchundi R, Neira R, Valdivia S (2011). Tau cleavage at D421 by caspase-3 is induced in neurons and astrocytes infected with Herpes Simplex Virus Type 1. *Journal of Alzheimer’s Disease*.

[110] Zhang Q, Zhang X, Sun A (2009). Truncated tau at D421 is associated with neurodegeneration and tangle formation in the brain of Alzheimer transgenic models. *Acta Neuropathologica*.

[111] Gamblin TC, Chen F, Zambrano A (2003). Caspase cleavage of tau: Linking amyloid and neurofibrillary tangles in Alzheimer’s disease. *Proceedings of the National Academy of Sciences of the United States of America*.

[112] Cho JH, Johnson GVW (2004). Glycogen synthase kinase 3*β* induces caspase-cleaved tau aggregation in situ. *Journal of Biological Chemistry*.

[113] De Calignon A, Fox LM, Pitstick R (2010). Caspase activation precedes and leads to tangles. *Nature*.

[114] Saito KI, Elce JS, Hamos JE, Nixon RA (1993). Widespread activation of calcium-activated neutral proteinase (calpain) in the brain in Alzheimer disease: a potential molecular basis for neuronal degeneration. *Proceedings of the National Academy of Sciences of the United States of America*.

[115] Rao MV, Mohan PS, Peterhoff CM (2008). Marked calpastatin (CAST) depletion in Alzheimer’s disease accelerates cytoskeleton disruption and neurodegeneration: neuroprotection by CAST overexpression. *Journal of Neuroscience*.

[116] Reinecke JB, DeVos SL, McGrath JP (2011). Implicating calpain in tau-mediated toxicity in vivo. *PLoS One*.

[117] Canu N, Dus L, Barbato C (1998). Tau cleavage and dephosphorylation in cerebellar granule neurons undergoing apoptosis. *Journal of Neuroscience*.

[118] Park SY, Ferreira A (2005). The generation of a 17 kDa neurotoxic fragment: an alternative mechanism by which tau mediates *β*-amyloid-induced neurodegeneration. *Journal of Neuroscience*.

[119] David DC, Layfield R, Serpell L, Narain Y, Goedert M, Spillantini MG (2002). Proteasomal degradation of tau protein. *Journal of Neurochemistry*.

[120] Lee BH, Lee MJ, Park S (2010). Enhancement of proteasome activity by a small-molecule inhibitor of USP14. *Nature*.

[121] Hutton M, Lendon CL, Rizzu P (1998). Association of missense and 5′-splice-site mutations in tau with the inherited dementia FTDP-17. *Nature*.

[122] Spillantini MG, Murrell JR, Goedert M, Farlow MR, Klug A, Ghetti B (1998). Mutation in the tau gene in familial multiple system tauopathy with presenile dementia. *Proceedings of the National Academy of Sciences of the United States of America*.

[123] Poorkaj P (1998). Tau is a candidate gene for chromosome 17 frontotemporal dementia. *Annals of Neurology*.

[124] Goedert M, Jakes R (2005). Mutations causing neurodegenerative tauopathies. *Biochimica et Biophysica Acta*.

[125] Magnani E, Fan J, Gasparini L (2007). Interaction of tau protein with the dynactin complex. *EMBO Journal*.

[126] Hong M, Hong M, Zhukareva V (1998). Mutation-specific functional impairments in distinct tau isoforms of hereditary FTDP-17. *Science*.

[127] Hasegawa M, Smith MJ, Goedert M (1998). Tau proteins with FTDP-17 mutations have a reduced ability to promote microtubule assembly. *FEBS Letters*.

[128] Hasegawa M, Smith MJ, Iijima M, Tabira T, Goedert M (1999). FTDP-17 mutations N279K and S305N in tau produce increased splicing of exon 10. *FEBS Letters*.

[129] Pickering-Brown SM, Baker M, Nonaka T (2004). Frontotemporal dementia with Pick-type histology associated with Q336R mutation in the tau gene. *Brain*.

[130] D’Souza I, Poorkaj P, Hong M (1999). Missense and silent tau gene mutations cause frontotemporal dementia with parkinsonism-chromosome 17 type, by affecting multiple alternative RNA splicing regulatory elements. *Proceedings of the National Academy of Sciences of the United States of America*.

[131] Nacharaju P, Lewis J, Easson C (1999). Accelerated filament formation from tau protein with specific FTDP-17 missense mutations. *FEBS Letters*.

[132] Goedert M, Jakes R, Crowther RA (1999). Effects of frontotemporal dementia FTDP-17 mutations on heparin-induced assembly of tau filaments. *FEBS Letters*.

[133] Gamblin TC, King ME, Dawson H (2000). In vitro polymerization of tau protein monitored by laser light scattering: method and application to the study of FTDP-17 mutants. *Biochemistry*.

[134] Barghorn S, Zheng-Fischhofer Q, Ackmann M (2000). Structure, microtubule interactions, and paired helical filament aggregation by tau mutants of frontotemporal dementias. *Biochemistry*.

[135] Von Bergen M, Barghorn S, Li L (2001). Mutations of Tau protein in frontotemporal dementia promote aggregation of paired helical filaments by enhancing local *β*-structure. *Journal of Biological Chemistry*.

[136] Grover A, England E, Baker M (2003). A novel tau mutation in exon 9 (1260V) causes a four-repeat tauopathy. *Experimental Neurology*.

[137] Neumann M, Diekmann S, Bertsch U, Vanmassenhove B, Bogerts B, Kretzschmar HA (2005). Novel G335V mutation in the tau gene associated with early onset familial frontotemporal dementia. *Neurogenetics*.

[138] DeTure M, Ko LW, Easson C, Yen SH (2002). Tau Assembly in inducible transfectants expressing wild-type or FTDP-17 tau. *American Journal of Pathology*.

[139] Noble W, Planel E, Zehr C (2005). Inhibition of glycogen synthase kinase-3 by lithium correlates with reduced tauopathy and degeneration in vivo. *Proceedings of the National Academy of Sciences of the United States of America*.

[140] Le Corre S, Klafki HW, Plesnila N (2006). An inhibitor of tau hyperphosphorylation prevents severe motor impairments in tau transgenic mice. *Proceedings of the National Academy of Sciences of the United States of America*.

[141] Alonso ADC, Mederlyova A, Novak M, Grundke-Iqbal I, Iqbal K (2004). Promotion of hyperphosphorylation by frontotemporal dementia tau mutations. *Journal of Biological Chemistry*.

[142] Alonso ADC, Zaidi T, Novak M, Grundke-Iqbal I, Iqbal K (2001). Hyperphosphorylation induces self-assembly of *τ* into tangles of paired helical filaments/straight filaments. *Proceedings of the National Academy of Sciences of the United States of America*.

[143] Goedert M, Satumtira S, Jakes R (2000). Reduced binding of protein phosphatase 2A to tau protein with frontotemporal dementia and parkinsonism linked to chromosome 17 mutations. *Journal of Neurochemistry*.

[144] Grover A, Houlden H, Baker M (1999). 5’ splice site mutations in tau associated with the inherited dementia FTDP-17 affect a stem-loop structure that regulates alternative splicing of exon 10. *Journal of Biological Chemistry*.

[145] Varani L, Hasegawa M, Spillantini MG (1999). Structure of tau exon 10 splicing regulatory element RNA and destabilization by mutations of frontotemporal dementia and parkinsonism linked to chromosome 17. *Proceedings of the National Academy of Sciences of the United States of America*.

[146] Donahue CP, Muratore C, Wu JY, Kosik KS, Wolfe MS (2006). Stabilization of the tau exon 10 stem loop alters pre-mRNA splicing. *Journal of Biological Chemistry*.

[147] Jiang Z, Cote J, Kwon JM, Goate AM, Wu JY (2000). Aberrant splicing of tau pre-mRNA caused by intronic mutations associated with the inherited dementia frontotemporal dementia with parkinsonism linked to chromosome 17. *Molecular and Cellular Biology*.

[148] D’Souza I, Schellenberg GD (2000). Determinants of 4-repeat tau expression. Coordination between enhancing and inhibitory splicing sequences for exon 10 inclusion. *Journal of Biological Chemistry*.

[149] D’Souza I, Schellenberg GD (2002). tau exon 10 expression involves a bipartite intron 10 regulatory sequence and weak 5′ and 3′ splice sites. *Journal of Biological Chemistry*.

[150] Jiang Z, Tang H, Havlioglu N (2003). Mutations in tau gene exon 10 associated with FTDP-17 alter the activity of an exonic splicing enhancer to interact with Tra2*β*. *Journal of Biological Chemistry*.

[151] Ludolph AC, Kassubek J, Landwehrmeyer BG (2009). Tauopathies with parkinsonism: Clinical spectrum, neuropathologic basis, biological markers, and treatment options. *European Journal of Neurology*.

[152] Pankratz N, Wilk JB, Latourelle JC (2009). Genomewide association study for susceptibility genes contributing to familial Parkinson disease. *Human Genetics*.

[153] Simon-Sanchez J (2009). Genome-wide association study reveals genetic risk underlying Parkinson's disease. *Nature Genetics*.

[154] Satake W, Nakabayashi Y, Mizuta I (2009). Genome-wide association study identifies common variants at four loci as genetic risk factors for Parkinson’s disease. *Nature Genetics*.

[155] Edwards TL, Scott WK, Almonte C (2010). Genome-Wide association study confirms SNPs in SNCA and the MAPT region as common risk factors for parkinson disease. *Annals of Human Genetics*.

[156] Tobin JE, Latourelle JC, Lew MF (2008). Haplotypes and gene expression implicate the MAPT region for Parkinson disease: the GenePD Study. *Neurology*.

[157] McKee AC, Cantu RC, Nowinski CJ (2009). Chronic traumatic encephalopathy in athletes: Progressive tauopathy after repetitive head injury. *Journal of Neuropathology and Experimental Neurology*.

[158] Duff K, Knight H, Refolo LM (2000). Characterization of pathology in transgenic mice over-expressing human genomic and cDNA tau transgenes. *Neurobiology of Disease*.

[159] Dawson HN, Cantillana V, Chen L, Vitek MP (2007). The tau N279K exon 10 splicing mutation recapitulates frontotemporal dementia and Parkinsonism linked to chromosome 17 tauopathy in a mouse model. *Journal of Neuroscience*.

[160] Rocher AB, Crimins JL, Amatrudo JM (2010). Structural and functional changes in tau mutant mice neurons are not linked to the presence of NFTs. *Experimental Neurology*.

[161] Paquet D, Schmid B, Haass C (2010). Transgenic zebrafish as a novel animal model to study tauopathies and other neurodegenerative disorders in vivo. *Neurodegenerative Diseases*.

[162] Mazanetz MP, Fischer PM (2007). Untangling tau hyperphosphorylation in drug design for neurodegenerative diseases. *Nature Reviews Drug Discovery*.

[163] Serenó L, Coma M, Rodríguez M (2009). A novel GSK-3*β* inhibitor reduces Alzheimer’s pathology and rescues neuronal loss in vivo. *Neurobiology of Disease*.

[164] Del Ser T, Steinwachs KC, Gertz HJ (2012). Treatment of Alzheimer's disease with the GSK-3 inhibitor tideglusib: a Pilot study. *Journal of Alzheimer's Disease*.

[165] Hu S, Begum AN, Jones MR (2009). GSK3 inhibitors show benefits in an Alzheimer’s disease (AD) model of neurodegeneration but adverse effects in control animals. *Neurobiology of Disease*.

[166] Tsai LH, Takahashi T, Caviness VS, Harlow E (1993). Activity and expression pattern of cyclin-dependent kinase 5 in the embryonic mouse nervous system. *Development*.

[167] Brunden KR, Trojanowski JQ, Lee VMY (2009). Advances in tau-focused drug discovery for Alzheimer’s disease and related tauopathies. *Nature Reviews Drug Discovery*.

[168] Crowe A, Huang W, Ballatore C (2009). Identification of aminothienopyridazine inhibitors of tau assembly by quantitative high-throughput screening. *Biochemistry*.

[169] Staff RT, Ahearn TS, Murray AD (2008). Tau aggregation inhibitor (TAI) therapy with rember arrests the trajectory of rCBF decline in brain regions affected by tau pathology in mild to moderate Alzheimer's disease. *Alzheimer's & Dementia*.

[170] O'Leary JC, Li Q, Marinec P (2010). Phenothiazine-mediated rescue of cognition in tau transgenic mice requires neuroprotection and reduced soluble tau burden. *Molecular Neurodegeneration*.

[171] Sawaya MR, Sambashivan S, Nelson R (2007). Atomic structures of amyloid cross-*β* spines reveal varied steric zippers. *Nature*.

[172] Sievers SA, Karanicolas J, Chang HW (2011). Structure-based design of non-natural amino-acid inhibitors of amyloid fibril formation. *Nature*.

[173] Zhang H, Burrows F (2004). Targeting multiple signal transduction pathways through inhibition of Hsp90. *Journal of Molecular Medicine*.

[174] Dickey CA, Yue M, Lin WL (2006). Deletion of the ubiquitin ligase CHIP leads to the accumulation, but not the aggregation, of both endogenous phospho- and caspase-3-cleaved tau species. *Journal of Neuroscience*.

[175] Dickey CA, Dunmore J, Lu B (2006). HSP induction mediates selective clearance of tau phosphorylated at proline-directed Ser/Thr sites but not KXGS (MARK) sites. *FASEB Journal*.

[176] Dickey CA, Kamal A, Lundgren K (2007). The high-affinity HSP90-CHIP complex recognizes and selectively degrades phosphorylated tau client proteins. *Journal of Clinical Investigation*.

[177] Luo W, Dou F, Rodina A (2007). Roles of heat-shock protein 90 in maintaining and facilitating the neurodegenerative phenotype in tauopathies. *Proceedings of the National Academy of Sciences of the United States of America*.

[178] Mizushima N, Levine B, Cuervo AM, Klionsky DJ (2008). Autophagy fights disease through cellular self-digestion. *Nature*.

[179] Hara T, Nakamura K, Matsui M (2006). Suppression of basal autophagy in neural cells causes neurodegenerative disease in mice. *Nature*.

[180] Komatsu M, Waguri S, Chiba T (2006). Loss of autophagy in the central nervous system causes neurodegeneration in mice. *Nature*.

[181] Ravikumar B, Vacher C, Berger Z (2004). Inhibition of mTOR induces autophagy and reduces toxicity of polyglutamine expansions in fly and mouse models of Huntington disease. *Nature Genetics*.

[182] Majumder S, Richardson A, Strong R, Oddo S (2011). Inducing autophagy by rapamycin before, but not after, the formation of plaques and tangles ameliorates cognitive deficits. *PLoS One*.

[183] Schaeffer V, Goedert M Stimulation of autophagy is neuroprotective in a mouse model of human tauopathy.

[184] Krishnamurthy PK, Sigurdsson EM (2011). Therapeutic applications of antibodies in non-infectious neurodegenerative diseases. *New Biotechnology*.

[185] Asuni AA, Boutajangout A, Quartermain D, Sigurdsson EM (2007). Immunotherapy targeting pathological tau conformers in a tangle mouse model reduces brain pathology with associated functional improvements. *Journal of Neuroscience*.

[186] Boutajangout A, Quartermain D, Sigurdsson EM (2010). Immunotherapy targeting pathological tau prevents cognitive decline in a new tangle mouse model. *Journal of Neuroscience*.

[187] Boimel M, Grigoriadis N, Lourbopoulos A, Haber E, Abramsky O, Rosenmann H (2010). Efficacy and safety of immunization with phosphorylated tau against neurofibrillary tangles in mice. *Experimental Neurology*.

[188] Troquier L, Caillierez R, Burnouf S (2012). Targeting phospho-Ser422 by active Tau Immunotherapy in the THYTau22 mouse model: a suitable therapeutic approach. *Current Alzheimer Research*.

[189] Boutajangout A, Ingadottir J, Davies P, Sigurdsson EM (2011). Passive immunization targeting pathological phospho-tau protein in a mouse model reduces functional decline and clears tau aggregates from the brain. *Journal of Neurochemistry*.

[190] Chai X (2011). Passive immunization with anti-Tau antibodies in two transgenic models: reduction of Tau pathology and delay of disease progression. *Journal of Biological Chemistry*.

[191] Kalbfuss B, Mabon SA, Misteli T (2001). Correction of alternative splicing of Tau in frontotemporal dementia and parkinsonism linked to chromosome 17. *Journal of Biological Chemistry*.

[192] Peacey E, Rodriguez L, Liu Y, Wolfe MS (2012). Targeting a pre-mRNA structure with bipartite antisense molecules modulates tau alternative splicing. *Nucleic Acids Research*.

[193] Varani L, Spillantini MG, Goedert M, Varani G (2000). Structural basis for recognition of the RNA major groove in the tau exon 10 splicing regulatory element by aminoglycoside antibiotics. *Nucleic Acids Research*.

[194] Donahue CP, Ni J, Rozners E, Glicksman MA, Wolfe MS (2007). Identification of tau stem loop RNA stabilizers. *Journal of Biomolecular Screening*.

[195] Liu Y, Peacey E, Dickson J (2009). Mitoxantrone analogues as ligands for a stem-loop structure of tau Pre-mRNA. *Journal of Medicinal Chemistry*.

[196] Zheng S, Chen Y, Donahue CP, Wolfe MS, Varani G (2009). Structural basis for stabilization of the Tau pre-mRNA splicing regulatory element by novantrone (mitoxantrone). *Chemistry and Biology*.

[197] Lopez-Senin P, Gomez-Pinto I, Grandas A, Marchan V (2011). Identification of ligands for the Tau exona 10 splicing regulatory element RNA by using dynamic combinatorial chemistry. *Chemistry*.

[198] Lu PJ, Wulf G, Zhou XZ, Davies P, Lu KP (1999). The prolyl isomerase Pin1 restores the function of Alzheimer-associated phosphorylated tau protein. *Nature*.

[199] Nakamura K, Greenwood A, Binder L (2012). Proline isomer-specific antibodies reveal the early pathogenic tau conformation in Alzheimer's disease. *Cell*.

[200] Mattson MP (2010). Acetylation unleashes protein demons of dementia. *Neuron*.

[201] Min SW, Cho SH, Zhou Y (2010). Acetylation of tau inhibits its degradation and contributes to tauopathy. *Neuron*.

[202] Zhang B, Maiti A, Shively S (2005). Microtubule-binding drugs offset tau sequestration by stabilizing microtubules and reversing fast axonal transport deficits in a tauopathy model. *Proceedings of the National Academy of Sciences of the United States of America*.

[203] Brunden KR, Zhang B, Carroll J (2010). Epothilone D improves microtubule density, axonal integrity, and cognition in a transgenic mouse model of tauopathy. *Journal of Neuroscience*.

[204] Zhang B (2012). The microtubule-stabilizing agent, epothilone D, reduces axonal dysfunction, neurotoxicity, cognitive deficits, and Alzheimer-like pathology in an interventional study with aged tau transgenic mice. *Journal of Neuroscience*.

[205] Barten DM, Fanara P, Andorfer C (2012). Hyperdynamic microtubules, cognitive deficits, and pathology are improved in tau transgenic mice with low doses of the microtubule-stabilizing agent BMS-241027. *Journal of Neuroscience*.

[206] Vulih-Shultzman I, Pinhasov A, Mandel S (2007). Activity-dependent neuroprotective protein snippet NAP reduces tau hyperphosphorylation and enhances learning in a novel transgenic mouse model. *Journal of Pharmacology and Experimental Therapeutics*.

[207] Matsuoka Y, Jouroukhin Y, Gray AJ (2008). A neuronal microtubule-interacting agent, NAPVSIPQ, reduces tau pathology and enhances cognitive function in a mouse model of Alzheimer’s disease. *Journal of Pharmacology and Experimental Therapeutics*.

[208] Shiryaev N, Jouroukhin Y, Giladi E (2009). NAP protects memory, increases soluble tau and reduces tau hyperphosphorylation in a tauopathy model. *Neurobiology of Disease*.

[209] Chakraborty C, Sarkar B, Hsu CH, Wen ZH, Lin CS, Shieh PC (2009). Future prospects of nanoparticles on brain targeted drug delivery. *Journal of Neuro-Oncology*.

